# Chiral Imidazolium Prolinate Salts as Efficient Synzymatic Organocatalysts for the Asymmetric Aldol Reaction

**DOI:** 10.3390/molecules26144190

**Published:** 2021-07-09

**Authors:** Raúl Porcar, Eduardo García-Verdugo, Belén Altava, Maria Isabel Burguete, Santiago V. Luis

**Affiliations:** 1 Departamento de Química Inorgánica y Orgánica, Universitat Jaume I, E-12071 Castellón de la Plana, Spain; rporcar@ccia.uned.es (R.P.); altava@uji.es (B.A.); burguete@uji.es (M.I.B.); 2 Departamento de Química Orgánica y Bio-Orgánica, Facultad de Ciencias, UNED, E-28040 Madrid, Spain

**Keywords:** aldol reaction, synzymes, homogeneous catalysis, organocatalysis, ionic liquids, imidazolium salts

## Abstract

Chiral imidazolium l-prolinate salts, providing a complex network of supramolecular interaction in a chiral environment, have been studied as synzymatic catalytic systems. They are demonstrated to be green and efficient chiral organocatalysts for direct asymmetric aldol reactions at room temperature. The corresponding aldol products were obtained with moderate to good enantioselectivities. The influence of the presence of chirality in both the imidazolium cation and the prolinate anion on the transfer of chirality from the organocatalyst to the aldol product has been studied. Moreover, interesting *match*/*mismatch* situations have been observed regarding configuration of chirality of the two components through the analysis of results for organocatalysts derived from both enantiomers of prolinate (*R*/*S*) and the *trans/cis* isomers for the chiral fragment of the cation. This is associated with differences in the corresponding reaction rates but also to the different tendencies for the formation of aggregates, as evidenced by nonlinear effects studies (NLE). Excellent activities, selectivities, and enantioselectivities could be achieved by an appropriate selection of the structural elements at the cation and anion.

## 1. Introduction

Asymmetric organocatalysis has become an important area of research in recent years for the preparation of chiral products, being an efficient alternative to enantioselective metal-based catalysis [1,2,3]. Since List and Barbas III reported the direct aldol reaction catalyzed by l-proline [4,5], a variety of different organocatalysts have been evaluated for this reaction, but l-proline derivatives still represent some of the best catalysts in this regard [6,7] and have been used successfully in a variety of other reactions [8]. The aldol reaction continues to be a key carbon–carbon bond-forming reaction, allowing the preparation of enantiomerically enriched *β*-hydroxy ketones, which are important building blocks for the synthesis of structurally complex molecules with biological activity, and numerous efforts are devoted to improving both yields and stereoselectivities for this reaction [9,10,11].

In recent years, room temperature ionic liquids (RTILs) have been intensively studied for the development of ‘‘green’’ synthetic processes [12,13,14,15]. Their modular character represents a key feature and facilitates introducing chirality elements into their structure. Thus, the design and synthesis of chiral ionic liquids (CILs) opens the possibility of developing new chiral media for application in separation technologies [16,17], for their use as chiral media in asymmetric synthesis, or as new enantioselective organocatalysts [18,19]. As in the case of ILs, CILs present, in general, a high degree of supramolecular organization [20], which can be exploited to enhance the resulting chirality transfer when used as chiral media, chiral additives, or organocatalysts in enantioselective processes [21]. In this regard, imidazolium-based CILs are relatively simple to prepare and have been studied in different organic transformations, including aldol reactions [22,23,24,25]. Many examples of organocatalytic CILs belong to the so-called “Ion-Tagged Ionic Liquids”, in which the chiral fragment is attached to an “onium” fragment (i.e., imidazolium) through an inert spacer [25,26,27]; although, the opposite approach is also possible—introducing the chiral organocatalytic fragment in the counteranion [28,29].

Recently, a variety of imidazolium ILs, including chiral and nonchiral compounds, have been studied as efficient reaction media or additives for the aldol reaction catalyzed by l-proline, with the results highlighting the importance of the involved supramolecular noncovalent interactions [30,31,32]. These results suggested the presence of well-defined supramolecular structures based on the correct disposition, including the correct configuration, of the functional elements located in the different components assembled. This situation mimics the one found in the active centers of enzymes and represents the key element defining synzymatic catalysts [33,34,35].

Following our previous contributions in the field of synzymes [36,37], and to better understand the phenomena taking place at the molecular and supramolecular levels in enantioselective aldol processes, as well as to advance in the rationalization of the design vectors for this kind of organocatalyst, here, we present the preparation of a series of chiral imidazolium cations containing prolinate as the counteranion and their study as organocatalysts for the aldol reaction. The resulting CILs contain chiral elements in both the anion and the cation, and the results show that both components are relevant for the enhancement in chirality transfer, with the observation of interesting *match/mismatch* arrangements for the configuration being present in both components.

## 2. Results and Discussion

**Figure 1:**
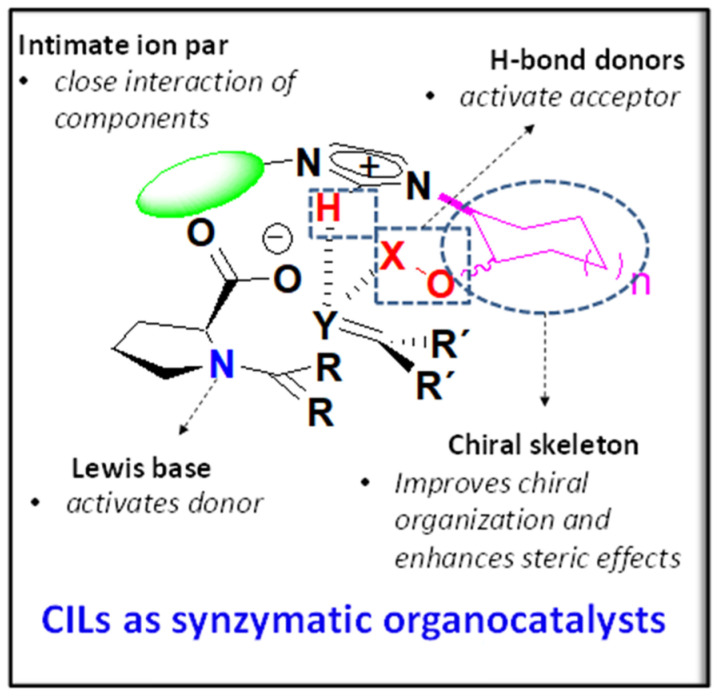
Main structural elements considered in the design of chiral imidazolium prolinates for their implementation as organocatalysts for aldol processes.

The general structure of the considered imidazolium prolinates is presented in Figure 1, highlighting the main structural elements that were considered relevant in their design. Such features are expected to implement supramolecular interactions, leading to the formation of the corresponding supramolecular complexes and the selective stabilization of the transition state for an enantioselective aldol reaction. A complex network of noncovalent forces such as ionic interactions, hydrogen bonds, Van der Waals forces, cation-π interactions, etc. can be implemented in those structures.

**Figure 2:**
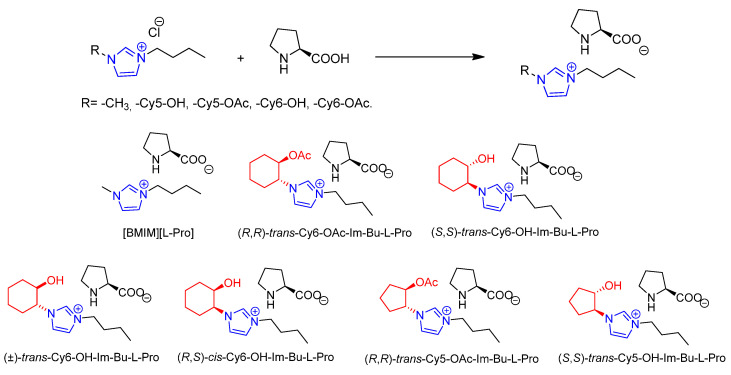
Preparation of chiral imidazolium prolinates through anion metathesis and structures for the CILs studied in this work (only l-prolinates shown).

The synthesis of those prolinates was carried out by a chloride anion exchange with l-proline (Figure 2). The starting chiral imidazolium chlorides were prepared as previously reported [38,39]. This simple metathesis process allowed the preparation—with excellent yields and purity and without needing any chromatographic purification—of a variety of organocatalysts. They differed in the relative configuration of the stereogenic centers in the imidazolium cation, in the presence of hydrogen bond donor (HBD) or hydrogen bond acceptor (HBA) groups in the chiral fragment, and in the size of the cycloalkyl ring in this fragment. The compound [BMIM][l-Pro] was also prepared from [BMIM][Cl], as an analogue displaying a nonchiral cation, for comparison purposes. In the same way, the diastereomeric salts (not shown in Figure 2) could also be prepared using d-proline. All these compounds were liquid at room temperature (RTILs). Although, in the case of [BMIM], the exchange of chloride by l-prolinate led to a significant reduction in the melting temperature (−33 °C vs. 48 °C, T_m_onset as obtained by DSC) the change was less relevant for the salts derived from the chiral imidazolium cations. Thus, for (*R*,*R*)-*trans*-Cy6-OAc-Im-Bu-, the change was from 19 °C to 2 °C, while for (*S*,*S*)-*trans*-Cy6-OH-Im-Bu-, the change was from 3 to 4 °C. In this last case, replacing l-prolinate by d-prolinate led to a similar behavior (T_m_onset = 2 °C).

**Figure 3:**
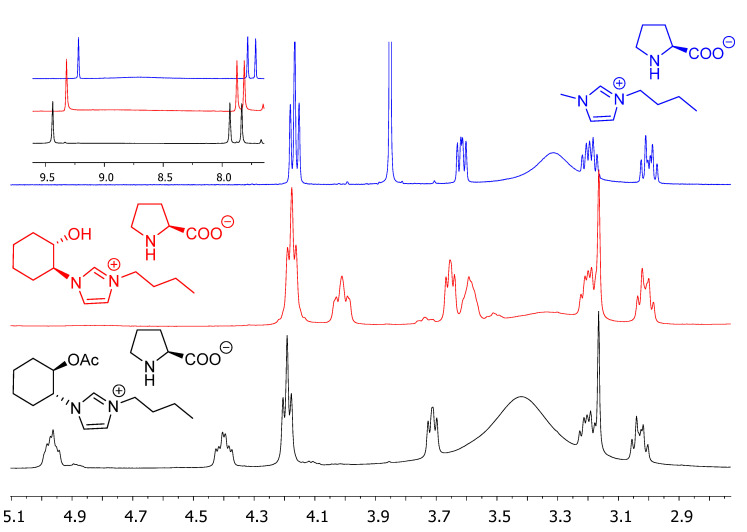
Partial ^1^H-NMR in DMSO-d_6_ for some imidazolium prolinates derived from l-proline.

The ^1^H-NMR spectra of these compounds in DMSO-*d*_6_ revealed the presence of proton signals corresponding to the prolinate anion as well as some interesting differences highlighting the importance of the structural parameters considered in the structure and noncovalent interactions present in these salts. Some illustrative examples are presented in Figure 3 (see also ESI, Figures S1–S5). Thus, signals corresponding to protons C2-H, C4-H, and C5-H of the imidazolium ring are shifted downfield in (*S*,*S*)-*trans*-Cy6-OH-Im-Bu-l-Pro (9.32, 7.87, 7.80 ppm) and (*R*,*R*)-*trans*-Cy6-OAc-Im-Bu-l-Pro (9.44, 7.95, 7.90 ppm) relative to [BMIM][l-Pro] (9.22, 7.81, 7.70 ppm), suggesting a more intense interaction of the anion with these relatively acidic protons. In the case of the anion, the signal for the stereogenic CH appears at 3.61 ppm in [BMIM][l-Pro], while it is slightly more unshielded in (*S*,*S*)-*trans*-Cy6-OH-Im-Bu-l-Pro (3.65 ppm) and (*R*,*R*)-*trans*-Cy6-OAc-Im-Bu-l-Pro (3.71 ppm). Overall, this also suggests the presence of a stronger ion pair in the case of the salt (*R*,*R*)-*trans*-Cy6-OAc-Im-Bu-l-Pro.

**Figure 4:**
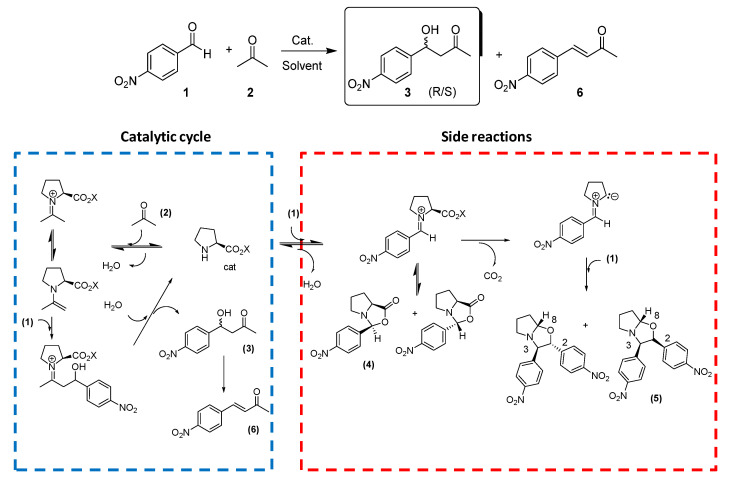
Benchmark aldol reaction between *p*-nitrobenzaldehyde (**1**) and acetone (**2**). General mechanisms for the formation of aldol and side products in the aldol reaction catalyzed by l-proline derivatives (adapted from Ref. [38]).

The room temperature aldol reaction of *p*-nitrobenzaldehyde with acetone was selected as the benchmark reaction to assess the organocatalytic potential of the prepared imidazolium salts (Figure 4) [40,41]. The initial experiments were carried out at room temperature, using toluene as the solvent, as this was expected to favor the formation of an intimate ion pair between the cation and the l-prolinate anion, and using a 40% molar loading of the catalyst and a 10:1 molar ratio acetone:aldehyde. Two different salts were firstly evaluated to analyze the influence of the hydroxy or ester fragments in the chiral moiety of the cation: (*R*,*R*)-*trans*-Cy6-OAc-Im-Bu-l-Pro and (*S*,*S*)-*trans*-Cy6-OH-Im-Bu-l-Pro (Table 1). Under the same conditions (Table 1, entry 1), l-proline only afforded a low yield of the aldol product, which can be associated to its low solubility in this solvent. This was solved with the use of the two selected chiral salts that afforded higher yields and maintained very good selectivities. Besides, significantly higher enantioselectivities were obtained (up to 70% ee, *R* major enantiomer, entry 4, Table 1). It must be noted that the yields and enantioselectivities were also higher than those obtained for [BMIM][l-Pro] (43% yield, 60% ee, entry 2, Table 1).

A second set of experiments were carried out in DMSO-d_6_, again using an excess (10 equiv.) of acetone over aldehyde and a 40% molar loading of the catalyst (0.02 M concentration) (Table 2). This allowed monitoring the progress of the reaction by ^1^H-NMR spectroscopy at room temperature. Under these conditions, higher conversions were obtained after 24 h, but the formation of significant amounts of side products was found in most cases, leading to low yields of the desired products. Analysis of the corresponding ^1^H-NMR spectra allowed us to identify some of those products, in agreement with the structures reported by Blackmond and coworkers [42]. They include oxazolidinones (**4**, Figure 4) formed by the reaction between proline and the aldehyde that subsequently can decarboxylate and react with an additional equivalent of aldehyde to produce 1-oxapyrrolizidines (**5**, Figure 4). An illustrative ^1^H-NMR spectrum from a crude reaction is shown in Figure S8. Formation of the side products took place almost instantly upon adding the aldehyde to the catalyst solution, and only increased slightly during the reaction. Carrying out the process in the absence of acetone allowed us to obtain those oxazolidinones and oxapyrrolizidines as the major products.

**Figure 5:**
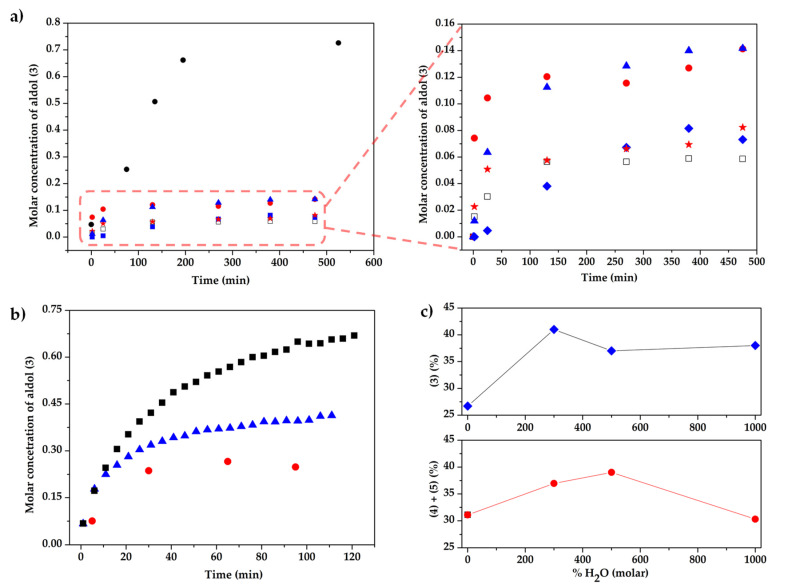
(**a**) Evolution of the concentration of the aldol product (**3**) with time for the different systems studied in DMSO-d_6_ (aldehyde(**1**):ketone(**2**) = 1:10) Catalytic systems: l-proline (★); [BMIM][l-Pro] (□); (±)-*trans*-Cy6-OH-Im-Bu-l-Pro (♦); (*S*,*S*)-*trans*-Cy6-OH-Im-Bu-l-Pro (●); (*R*,*R*)-*trans*-Cy6-OAc-Im-Bu-l-Pro (▲); inset: l-Pro:(*R*,*R*)-*trans*-Cy6-OAc-Im-Bu-Cl (1:4) (●). (**b**) Evolution of the concentration of **3** with time for the reaction catalyzed by [BMIM][l-Pro] in DMSO-d_6_ under different conditions; **1**:**2**:cat:H_2_O: 1:10:0.4:0 (●); 1:10:0.4:10 (▲); 1:30:0.4:10 (■). (**c**) Effect of water on the yield and enantioselectivity of the aldol product (top) and on the amount of side products ((**4**) and (**5**)) formed (bottom) after 24 h for the reaction catalyzed by [BMIM][l-Pro] (**1**:**2**:cat = 1:10:0.4, water molar excess relative to **1**, DMSO-*d*_6_).

The kinetic profiles obtained provided additional information. Figure 5a shows the evolution of the concentration of the aldol product (**3**) with time for the different systems studied. Thus, the prolinate salts (*R*,*R*)-*trans*-Cy6-OAc-Im-Bu-l-Pro and (*S*,*S*)-*trans*-Cy6-OH-Im-Bu-l-Pro were slightly more active than l-proline, [BMIM][l-Pro] and the catalyst based on the racemic cation ((±)-*trans*-Cy6-OH-Im-Bu-l-Pro). This agrees well with the yields presented in Table 2 and suggests that the prolinate salts of the two enantiomers of *trans*-Cy6-OH-Im-Bu-l-Pro can display significantly different activities, with the (*R*,*R*) enantiomer being catalytically less active. For comparison purposes, the left side in Figure 5a also includes the kinetic profile for the reaction catalyzed by l-proline in the presence of four equivalents of the CIL (*R*,*R*)-*trans*-Cy6-OAc-Im-Bu-Cl. In this case, a significant suppression of the formation of the side products was observed, leading to a quantitative conversion of the aldehyde and a 73% yield of the aldol product (Table 2, entry 6), in agreement with results obtained previously for the solventless reaction studied using mixtures of CILs and l-proline [31].

The enantioselectivities observed are presented in the last column in Table 2. In all cases, the presence of an imidazolium cation significantly increased the enantioselectivity achieved by l-proline (36% ee). Besides, the presence of chirality in the imidazolium cation clearly improved the resulting ee values (from 56% for [BMIM] to up to 81%). The presence in the cyclohexyl chiral fragment attached to the imidazolium of the acetate group, instead of the hydroxy group, was beneficial in this regard (Table 2, compare entries 3 and 5). The use of the racemic cation was reflected in a decrease of the enantioselectivity achieved in comparison with the enantiopure counterpart (Table 2, entries 3 and 4). Interestingly, the enantioselectivity afforded by the mixture between l-proline and (*R*,*R*)-*trans*-Cy6-OAc-Im-Bu-Cl provided a lower ee value than the l-prolinate of (*R*,*R*)-*trans*-Cy6-OAc-Im-Bu-Cl under the same conditions (Table 2, entries 5 and 6).

The controlled addition of water has been reported to reduce some of the side reactions in this kind of aldol reactions [42,43,44,45]. This possibility was analyzed using initially [BMIM][l-Pro] as the catalytic system, adding acetone first to minimize oxazoline formation and using from 0 to 1000 equivalents of water relative to the aldehyde. The kinetic profiles for the formation of the aldol product **3** catalyzed by [BMIM][l-Pro] reveal that the presence of water accelerates the reaction (Figure 5b). As seen in Figure 5c, some improvement in the yields for **3** was observed, reaching values of up to 40%, although significant amounts of side products **4** and **5** were still formed (30–40%). The use of a large excess of acetone (30 equiv.), in the presence of 1000 equiv. of water allowed improving aldol yields up to 89%, with a considerable reduction in the side products (10%). The addition of water did not have, however, a significant influence on the enantiomeric excess obtained (67–70% ee). In contrast, the reaction using 30 equiv. of acetone, although more selective, afforded some decrease in the observed induction (60% ee). Similar patterns were observed for catalysts containing chiral imidazolium cations. The kinetic profiles were very similar in the presence of water (Figure S9) and yields for aldol **3** increased to 65–70% with the formation of less than 15% of side products **4** and **5**. Nevertheless, the ee values, being higher than those for [BMIM][l-Pro], were lower than under the conditions reported in Table 2 (i.e., 72% ee for (*R*,*R*)-*trans*-Cy6-OAc-Im-Bu-l-Pro).

In a third set of experiments, the same reaction was studied under solventless conditions, using a 1:10 *p*-nitrobenzaldehyde:acetone-*d*_6_ molar ratio and, again, a molar catalyst loading of 40%. Results in Table 3 show that quantitative conversions were achieved after 24 h, with excellent selectivities to the aldol **3** in most instances. The only side product detected was the enone **6** resulting from the dehydration of the aldol. Under these conditions, the use of [BMIM][l-Pro] provided a low enantiomeric excess (20% ee). In contrast, the use of l-prolinates from chiral imidazolium cations achieved significantly higher ee values. In most instances, they were in the 60–70% range. A detailed analysis of the influence of chiral hydroxy- or acetoxycycloalkyl moiety on enantioselectivity is not easy because the starting enantiopure amino alcohols or amino acetates are obtained through the enzymatic kinetic resolution of the racemic amino acetates, which provides accessibility only to some of the possible configurations [30]. Comparison between entries 2 and 6 as well as between entries 3 and 7 in Table 3 suggests that compounds containing cyclopentyl rings provide higher enantioselectivities than those with cyclohexyl rings, which can be assigned to the higher conformational flexibility of the six-membered rings. The most relevant observation, however, was that the selectivity achieved by organocatalysts containing 1-*β*-hydroxycyclohexyl-3-butyl-imidazolium cations depended greatly on the configuration of the C-OH carbon. A much less selective (79%) and enantioselective process (43% ee) was observed for (*S*,*S*)-*trans*-Cy6-OH-Im-Bu-l-Pro than for (*R*,*S*)-*cis*-Cy6-OH-Im-Bu-l-Pro (95% selectivity, 64% ee) (Table 3, entries 3 and 4). This was confirmed by the results for the racemic cation (±)-*trans*-Cy6-OH-Im-Bu (Table 3, entry 5). The selectivity (96%) and enantioselectivity (64%) for the prolinate of this racemic cation indicate that the (*R*,*R*)-*trans*-Cy6-OH-Im-Bu-l-Pro organocatalyst displays a high activity, selectivity, and enantioselectivity, suggesting the presence of a “*match/mismatch*” effect between the configuration of the C-OH carbon atom and the one of l-prolinate. Related “*match/mismatch*” effects have been reported for asymmetric catalytic systems containing two chiral components, as is the case in the use of chiral cocatalysts, chiral activators, or two chiral ligands in catalytic metal complexes [32,46,47,48,49,50].

**Figure 6:**
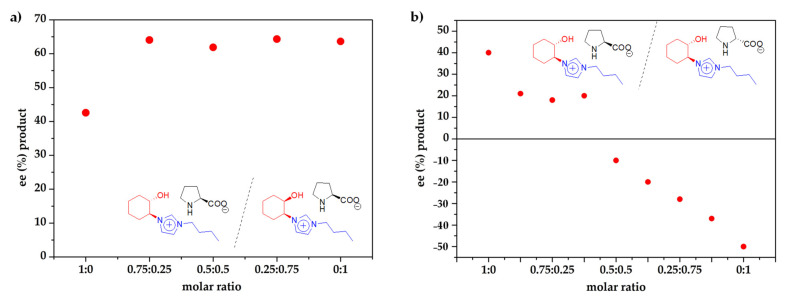
(**a**) Influence of the configuration at the C-OH carbon of the imidazolium cation on the enantioselectivity observed. (**b**) Influence of the configuration at the prolinate anion on the enantioselectivity observed towards the *R* aldol product. Conditions: rt, 23 h; 1:10 aldehyde(**1**):acetone(**2**) ratio; constant concentration of catalyst (0.55 M).

In order to analyze in more detail this “*match/mismatch*” effect, a similar reaction was carried out using different molar mixtures of the catalysts (*S*,*S*)-*trans*-Cy6-OH-Im-Bu-l-Pro and (*R*,*S*)-*cis*-Cy6-OH-Im-Bu-l-Pro (Table S1, Figure 6a). All mixtures were active with a quantitative conversion of the aldehyde after 23 h and providing the compound resulting from the dehydration of the aldol as the only detected side product, with the observation of a pronounced nonlinear effect (NLE) [51,52]. The presence of (*R*,*S*)-*cis*-Cy6-OH-Im-Bu-l-Pro, even as a minor component of the mixture, resulted in an excellent selectivity and increased enantiomeric excesses (>60). Both the formation of dimers or other aggregates and kinetic factors have been used to rationalize nonlinear effects [51,52], though the simpler explanation for a simple NLE, such as the one in Figure 6a, involves that the organocatalyst (*R*,*S*)-*cis*-Cy6-OH-Im-Bu-l-Pro is both more active and enantioselective than (*S*,*S*)-*trans*-OH-Cy6-Im-Bu-l-Pro, as found in other diastereomeric mixtures [53,54,55].

A better “*match*” between the configurations at the cation and the anion was achieved for the (*S*,*S*)-*trans*-Cy6-OH-Im-Bu cation when combined with the d-prolinate. The (*S*,*S*)-*trans*-Cy6-OH-Im-Bu-d-Pro salt afforded a significantly higher selectivity (96% vs. 79%) and enantioselectivity than the l-prolinate (50% ee vs. 40% ee with a reversal in the topicity of the aldol, the (*S*)-enantiomer being the major product). This confirms that the combination between the d-prolinate and the (*S*,*S*)-cation represents a better *match* than the l/(*S*,*S*) combination. When using different molar mixtures of the two salts with d- and l-prolinate, a complex nonlinear effect was observed (Figure 6b, Table S2). Such relationships have been often observed in diastereomeric systems and are associated to a complex aggregation behavior with the participation of homo- and/or heteroaggregates [56,57]. Interestingly, a 10% ee (*S*) was obtained for the equimolecular mixture, while for the region where (*S*,*S*)-*trans*-Cy6-OH-Im-Bu-l-Pro was the major component, the enantioselectivity remained essentially constant (ca. 20% ee towards the (*R*)-aldol) for the region where this salt represented ca. 60–90% of the mixture. These results suggest that (*S*,*S*)-*trans*-Cy6-OH-Im-Bu-l-Pro favors aggregation, leading to stable and less-reactive heteroaggregates. It is worth mentioning that for diastereomeric mixtures, homoaggregates can also have significantly different stabilities and activities [58].

Cyclohexanone represents a more demanding substrate for aldol reactions and is quite sensitive to environmental factors. Though the enantioselectivities achieved tend to be higher than with acetone, this ketone is less reactive and can provide four different products as a consequence of the formation of anti and syn diastereomers [30]. When the reaction was carried out in DMSO (1:10 aldehyde(**1**):ketone ratio), the results with (*R*,*R*)-*trans*-Cy6-OAc-Im-Bu-l-Pro and (*S*,*S*)-*trans*-Cy6-OH-Im-Bu-l-Pro were very similar to those with l-proline or with [BMIM][l-Pro]. Thus, *anti*:*syn* diastereoselectivities close to 45:55, with enantiomeric excesses of ca. 70% ee (*anti*) and 95% ee (*syn*), were observed (Table S3). When this solvent (DMSO) was substituted by [BMIM][NTF_2_], improved results were obtained, with aldol yields of 75–80%, *anti*:*syn* selectivities of ca. 65:35, and excellent enantioselectivites (ca. 98% ee for the *anti* products and ca. 90% ee for the *syn*). Finally, when the conditions optimized for acetone were used (1:10 aldehyde(**1**):ketone, in the absence of solvent), excellent yields were obtained for some of the prolinates assayed (Table 4). *Anti*:*syn* selectivities also were significantly enhanced, ranging from 75:25 to 90:10. Interestingly, the catalysts containing cyclopentyl fragments not only achieved quantitative yields, but also the highest selectivities (90:10) and enantioselectivities, reaching 96% ee (*anti*) and 79% ee (*syn*) when using (*S*,*S*)-*trans*-Cy5-OH-Im-Bu-l-Pro. It must be noted that for Cy5 catalysts, the topicity of the major enantiomer for the *syn* pair was reversed relative to the one found with Cy6 and [BMIM][l-Pro] catalysts, while the same topicity was obtained for the *anti* pair.

**Figure 7:**
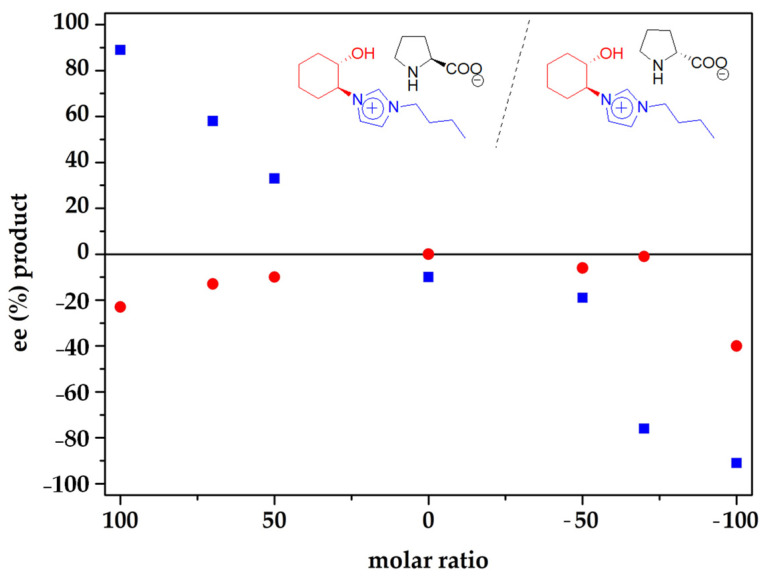
Influence of the configuration at the prolinate anion on the enantioselectivity observed for the *anti* (blue squares) and *syn* (red circles) pairs formed in the aldol reaction between *p*-nitrobenzaldehyde and cyclohexanone using different molar ratios of (*S*,*S*)-*trans*-Cy6-OH-Im-Bu-l-Pro:(*S*,*S*)-*trans*-Cy6-OH-Im-Bu-d-Pro (40% molar loading) at room temperature under solventless conditions: 27 h, 1:10 aldehyde(**1**):cyclohexanone ratio, constant concentration of catalyst (0.38 M).

In order to further analyze the former “*match/mismatch*” effects observed, the reaction was also studied using (*S*,*S*)-*trans*-Cy6-OH-Im-Bu-d-Pro. Under the same conditions used with (*S*,*S*)-*trans*-Cy6-OH-Im-Bu-l-Pro, in the absence of solvent, comparable results were obtained in terms of yield (88 vs. 80%) and *anti/syn* selectivity (80:20 vs. 82:18) and a similar value with topicity reversal was achieved for the enantioselectivity of the *anti* pair (−91 vs. 89% ee). The situation, however, was fully different when the selectivity of the *syn* pair was analyzed. No topicity reversal was observed in this case and the substitution of the l-prolinate by the d-prolinate led to an increase in the ee towards the same enantiomer (−40% vs. −23% ee). The enantioselectivities observed for different molar mixtures of (*S*,*S*)-*trans*-Cy6-OH-Im-Bu-l-Pro and (*S*,*S*)-*trans*-Cy6-OH-Im-Bu-d-Pro are presented in Figure 7 (see also Table S4) and show that, for the *anti* pair, a clear correlation exists between the l-prolinate/d-prolinate ratio and the observed ee. This relationship, however, is not fully linear; for instance, for a 50:50 ratio an ee value of −10 was obtained. For the *syn* pair, no clear correlation is observed with the l-prolinate/d-prolinate ratio in the catalytic mixture. The same major enantiomer is produced for all mixtures, indicating that the configuration of the prolinate anion is not the structural element defining the topicity of the major *syn* enantiomer and suggesting the presence of a complex aggregation behavior, which is also substrate dependent [59,60].

Finally, the scope of the aldol reaction between some additional aldehydes and cyclohexanone was explored with some of these catalytic systems using the optimized conditions: 1:10 aldehyde:ketone ratio, rt, solventless, 40% catalyst loading, 24 h (Table 5). Results show a clear improvement over those for l-proline. Only the least-activated aldehyde (4-methoxybenzaldehyde) provided lower yields, selectivities, and enantioselectivities than 4-nitrobenzaldehyde. For the other aldehydes, *anti*:*syn* selectivities ranged between 73:25 and 81:19, with enantioselectivities being clearly higher for the *anti* pair (up to 99% ee). Similar trends were observed when the aldol reaction between acetone and several aldehydes was studied (Table S4).

## 3. Materials and Methods

### 3.1. Experimental Section

All reagents were obtained from Sigma-Aldrich. Flash chromatography was performed using silica gel 60 (230–240 mesh). ^1^H-NMR and ^13^C-NMR experiments were obtained using a Varian INOVA 500 (^1^H, 500 MHz and ^13^C, 125 MHz) spectrometer. Chemical shifts are given in delta (*δ*, ppm) values and the coupling constants (J) in Hertz (Hz). High-performance liquid chromatography (HPLC) analyses were carried out on a Merck HITACHI LaChrom chromatograph with an UV detector at 254 nm, 210 nm, or 230 nm, using the chiral chromatography columns Daicel Chiralcel OJ, Daicel Chiralcel OD-H, or Chiralpak AD (25 cm × 4.6 mm I.D.).

### 3.2. General Procedure for the Preparation of Imidazolium Prolinates

To a solution of the corresponding imidazolium salt (1.21 mmol) in MeOH (5 mL), l- or d-proline was added (140.9 mg, 1.21 mmol). The resulting mixture was stirred at room temperature for 24–72 h. After this time, the solvent was removed by distillation under reduced pressure to obtain the corresponding imidazolium salts as whitish, viscous liquids.

### 3.3. General Procedure for the Aldol Reactions

Over a mixture of 0.4 mmol of the corresponding CIL (as organocatalyst) and 10 mmoles of acetone or cyclohexanone, 1 mmol of *p*-nitrobenzaldehyde was added. The resulting mixture was stirred for 20–24 h at room temperature, following the reaction by TLC (Hex:AcOEt, 2:1). After that time, 10 mL of chloroform were added, and the mixture was washed with deionized water (3 × 5 mL). The organic phase was dried with anhydrous magnesium sulphate and the solvent was evaporated under reduced pressure. The resulting crude was purified by flash chromatography on silica gel (Hex:AcOEt, 2:1). Details for the characterization of the different products and the HPLC methods used are given in the Supporting information.

## 4. Conclusions

Chiral room temperature ionic liquids derived from chiral imidazolium cations and prolinate anions can be efficiently used as organocatalysts for direct aldol reactions. The presence of chirality in both the cation and the anion not only increment the structural optimization vectors but allows the formation of spatially well-defined supramolecular complexes with the substrates and reaction intermediates in a truly synzymatic approach. The complex network of supramolecular interactions is able to selectively stabilize some of the intermediates and transition states leading to significant improvements in activity, selectivity, and particularly enantioselectivity, and to efficiently work under solventless conditions. Results were always improved over the use of l-Pro or its salts with nonchiral cations. Thus, for instance, in the case of the benchmark reaction between 4-nitrobenzaldehyde and acetone, the salt (*R*,*R*)-*trans*-Cy6-OAc-Im-Bu-l-Pro showed faster kinetics than other salts and, besides, the enantioselectivity was significantly improved relative to the ones observed for salts with achiral cations: 66% ee vs. 13% ee for [BMIM][l-Pro]. Important *match*/*mismatch* effects are present regarding the configurations of the anion and the cation. Such effects are not only associated with the supramolecular organization of the two components of the catalytic system alone and that of the complexes formed with the substrates and intermediates during the reaction, but also affect the formation of aggregates, as suggested by the observed nonlinear effects. Enantioselectivity was significantly enhanced when sterically more-demanding substrates are used, reaching, for instance, 96% ee for the major *anti* pair (90:10 *anti*:*syn* selectivity) in the case of cyclohexanone and 4-nitrobenzaldehyde ((*S*,*S*)-*trans*-Cy5-OH-Im-Bu-l-Pro) or 99% for the major *anti* pair (76:24 *anti*:*syn* selectivity) in the case of cyclohexanone and benzaldehyde ((*S*,*S*)-*trans*-Cy6-OH-Im-Bu-l-Pro). Thus, the high diversity achievable with these systems allows for simple fine-tuning of the optimal catalytic structure for each of the specific substrates considered.

## Figures and Tables

**Table 1:** Results for the aldol reaction between *p*-nitrobenzaldehyde and acetone with different imidazolium l-prolinates (40% catalyst loading) at room temperature in toluene ^1^.

Entry	Imidazolium Salts	Conversion (%) ^2^	Selectivity (%) ^2^	Yield (%) ^2^	ee (%) ^3^
1 ^4^	-	10	99	10	50
2	[BMIM][l-Pro]	45	95	43	60
3	(*R*,*R*)-*trans*-Cy6-OAc-Im-Bu-l-Pro	73	94	69	66
4	(*S*,*S*)-*trans*-Cy6-OH-Im-Bu-l-Pro	68	93	63	70

^1^ Conditions: 1:10:0.4 aldehyde(**1**):acetone(**2**):cat ratio, toluene (1 mL), rt, 44 h; constant concentration of catalyst (0.04 M). ^2^ Conversion, selectivity (percentage of aldol compounds in the products of transformation of the aldehyde), and aldol yield calculated by ^1^H-NMR in the crude of the reaction. ^3^ Enantiomeric excess calculated by HPLC for the *R* enantiomer (major peak) [ee = (peak area (*R*) − peak area (*S*)) × 100/total area (*R* + *S*)]. ^4^ l-proline was used as the catalyst (40%).

**Table 2:** Results for the aldol reaction between *p*-nitrobenzaldehyde and acetone with different imidazolium l-prolinates (40% catalyst loading) at room temperature in DMSO-d6 ^1^.

Entry	Catalyst	Conv. (%) ^2^	Yield (%) ^2^	Side Products (4 + 5)(%) ^3^	ee (%) ^4^
1 ^5^	l-Pro	70	9	32	36
2	[BMIM][l-Pro]	63	11	23	56
3	(*S*,*S*)-*trans*-Cy6-OH-Im-Bu-l-Pro	70	17	26	73
4	(*±*)-*trans*-Cy6-OH-Im-Bu-l-Pro	64	9	27	62
5	(*R*,*R*)-*trans*-Cy6-OAc-Im-Bu-l-Pro	80	17	32	81
6 ^6^	l-Pro + (*R*,*R*)-*trans*-Cy6-OAc-Im-Bu-Cl	99	73	14	70

^1^ Conditions: 1:10:0.4 aldehyde(**1**):acetone(**2**):cat ratio, DMSO-d_6_, rt, 24 h; constant concentration of catalyst (0.02 M). ^2^ Conversion, selectivity, and yield calculated by ^1^H-NMR in the crude of the reaction. ^3^ Overall molar percentage for the four side products (**4** + **5**) detected. ^4^ Enantiomeric excess calculated by HPLC for the *R* enantiomer (major peak) [ee = (peak area (*R*) − peak area (*S*)) × 100/total area (*R* + *S*)]. ^5^ l-proline was used as the catalyst (40%). ^6^ l-proline:(*R*,*R*)-*trans*-Cy6-OAc-Im-Bu-Cl (1:4).

**Table 3:** Results for the aldol reaction between *p*-nitrobenzaldehyde and acetone-*d*_6_ with different imidazolium l-prolinates (40% catalyst loading) at room temperature under solventless conditions ^1^.

Entry	Imidazolium Salts	Conversion (%) ^2^	Selectivity (%) ^2^	ee (%) ^3^
1	[BMIM][l-Pro]	>99	91	20
2	(*R*,*R*)-*trans*-Cy6-OAc-Im-Bu-l-Pro	>99	98	66
3	(*S*,*S*)-*trans*-Cy6-OH-Im-Bu-l-Pro	>99	79	43
4	(*R*,*S*)-*cis*-Cy6-OH-Im-Bu-l-Pro	>99	95	64
5	(±)-*trans*-Cy6-OH-Im-Bu-l-Pro	>99	96	64
6	(*R*,*R*)-*trans*-Cy5-OAc-Im-Bu-l-Pro	>99	90	68
7	(*S*,*S*)-*trans*-Cy5-OH-Im-Bu-l-Pro	>99	90	60

^1^ Conditions: 1:10 aldehyde(**1**):acetone-d_6_(**2**) ratio, rt, 24 h, constant concentration of catalyst (0.55 M). ^2^ Conversion, selectivity (percentage of aldol compounds in the products of transformation of the aldehyde), and aldol yield calculated by ^1^H-NMR in the crude of the reaction. ^3^ Enantiomeric excess calculated by HPLC for the enantiomer *R* (major peak) [ee = (peak area (*R*) − peak area (*S*)) × 100/total area (*R* + *S)*].

**Table 4:** Results for the aldol reaction between *p*-nitrobenzaldehyde and cyclohexanone using different prolinates (40% catalyst loading) at room temperature in the absence of solvent ^1^.

Entry	Catalyst	Yield (%) ^2^	Selectivity *anti*:*syn* ^2^	ee*_anti_* (%) ^3^	ee*_syn_* (%) ^3^
1	[BMIM][l-Pro]	86	86:14	90	−3
2	(*R*,*R*)-*trans*-Cy6-OAc-Im-Bu-l-Pro	79	75:25	86	−74
3	(*S*,*S*)-*trans*-Cy6-OH-Im-Bu-l-Pro	80	82:18	90	−23
4	(*R*,*R*)-*trans*-Cy5-OAc-Im-Bu-l-Pro	99	90:10	95	73
5	(*S*,*S*)-*trans*-Cy5-OH-Im-Bu-l-Pro	99	90:10	96	79

^1^ Conditions: 1:10 aldehyde(**1**):cyclohexanone ratio, rt, 27 h, constant concentration of catalyst (0.38 M). ^2^ Selectivity and yield calculated by ^1^H-NMR in the crude of the reaction. ^3^ Enantiomeric excess calculated by HPLC; the peak at [ee = (peak area (*B*) − peak area (*A*)) × 100/total area (*A* + *B*)], where peak (*A*) is the one displaying a shorter retention time in the HPLC chromatogram (for each *anti* or *syn* pair).

**Table 5:**
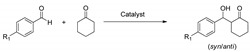
Results of the aldol reaction between different aldehydes and cyclohexanone at room temperature for 24 h ^1^.

Entry	R_1_	Catalyst	Yield (%) ^2^	Selectivity *anti*:*syn* ^2^	ee*_anti_* (%) ^3^	ee*_syn_* (%) ^3^
1	H	l-proline	78	64:36	41	n.d
		(*R*,*R*)-*trans*-Cy6-OAc-Im-Bu-l-Pro	89	76:24	88	19
		(*S*,*S*)-*trans*-Cy6-OH-Im-Bu-l-Pro	79	76:24	99	71
2	Cl	l-proline	63	63:37	54	n.d
		(*R*,*R*)-*trans*-Cy6-OAc-Im-Bu-l-Pro	95	81:19	93	31
		(*S*,*S*)-*trans*-Cy6-OH-Im-Bu-l-Pro	92	79:21	68	57
3	OMe	l-proline	-	-	-	-
		(*R*,*R*)-*trans*-Cy6-OAc-Im-Bu-l-Pro	61	69:31	86	47
		(*S*,*S*)-*trans*-Cy6-OH-Im-Bu-l-Pro	60	68:32	54	37
4	F	l-proline	38	64:36	65	60
		(*R*,*R*)-*trans*-Cy6-OAc-Im-Bu-l-Pro	90	77:23	92	51
		(*S*,*S*)-*trans*-Cy6-OH-Im-Bu-l-Pro	93	73:27	47	27

^1^ Conditions: 1:10 aldehyde:cyclohexanone ratio, rt, 27 h, constant concentration of catalyst (0.38 M). ^2^ Selectivity and yield calculated by ^1^H-NMR in the crude of the reaction. ^3^ Enantiomeric excess calculated by HPLC; the peak at [ee = (peak area (*B*) − peak area (*A*)) × 100/total area (*A* + *B*)], where peak (*A*) is the one displaying a shorter retention time in the HPLC chromatogram (for each *anti* or *syn* pair). n.d: not detected.

## Supplementary Materials

The following are available online. Characterization of imidazolium salts including Figures S1–S5. Additional catalytic experiments including Figures S8 and S9 and Tables S1–S4. Characterization of aldol products including Figures S10–S18 with HPLC chromatograms.

## Sample Availability

Samples of compounds are available from the authors.
