# Supplementary material for: Chiral Imidazolium Prolinate Salts as Efficient Synzymatic Organocatalysts for the Asymmetric Aldol Reaction
Source: Molecules. 2021 Jul 9;26(14):4190. doi: 10.3390/molecules26144190 (PMC8303523; doi:10.3390/molecules26144190)
Supplement: Supplementary file 1 [file molecules-26-04190-s001.zip › molecules-1274166-supplementary.pdf]

Supporting information

Chiral imidazolium prolinate salts as efficient synzymatic organocatalysts for the asymmetric aldol reaction

Raúl Porcar [1,2], Eduardo García-Verdugo [1]*, Belén Altava [1], M. Isabel Burguete [1] and Santiago V. Luis [1,*]

[1] Departamento de Química Inorgánica y Orgánica, Universitat Jaume I, E-12071, Castellón de la Plana (Spain); luiss@uji.es
[2] Departamento de Química Orgánica y Bio-Orgánica, Facultad de Ciencias, UNED, E-28040, Madrid (Spain); rporcar@ccia.uned.es
Correspondence: luiss@uji.es and cepeda@uji.es

Table of Contents

**1. Characterization of imidazolium salts**	**SI2**
**2. Additional catalytic experiments**	**SI14**
**3. Characterization of aldol products**	**SI17**

1. Characterization of imidazolium salts

1-((1*R*,2*R*)-2-acetoxycyclohexyl)-3-butyl-1H-imidazol-3-ium (*S*)-pyrrolidine-2-carboxylate [(*R*,*R*)-*trans*-Cy6-OAc-Im-Bu-L-Pro]

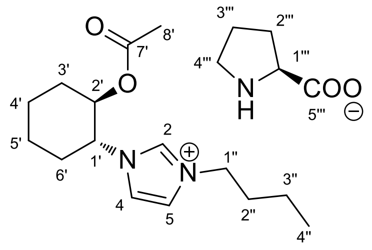

Gummy colorless solid
Empirical formula: $C_{20}H_{33}N_3O_4$
Molecular weight: 379.49 g/mol
Melting point: 2 ºC

IR (ATR): ν 3378, 3046, 2935, 2865, 1735, 1626, 1558, 1453, 1373, 1230, 1168, 1031, 959 cm^{-1}

^{1}H-NMR ($CDCl_3$, 500 MHz): δ 0.96 (t, 3H, *J* = 7.4 Hz, $H_{4''}$), 1.36 (td, 2H, *J* = 7.2, 14.6 Hz, $H_{3''}$), 1.43-1.62 (m, 3H, $H_{3'}$+2$H_{5'}$), 1.84-1.92 (m, 6H, 2$H_{4'}$+$H_{6'}$+2$H_{2''}$+$H_{3'''}$), 1.94 (s, 3H, $H_{8'}$), 1.96-2.04 (m, 1H, $H_{3'''}$), 2.07-2.19 (m, 2H, $H_{3'}$+$H_{2'''}$), 2.23-2.41 (m, 2H, $H_{6'}$+$H_{2'''}$), 3.28-3.41 (m, 2H, $H_{4'''}$), 4.05 (dd, 1H, *J* = 5.7, 8.7 Hz, $H_{1'''}$), 4.36 (dd, 1H, *J* = 7.0, 13.9 Hz, $H_{1''}$), 4.45 (dd, 1H, *J* = 7.0, 14.0 Hz, $H_{1''}$), 4.61 (d, 1H, *J* = 10.2 Hz, $H_{2'}$), 4.90-5.01 (m, 1H, $H_{1'}$), 7.21 (s, 1H, H_4), 7.26 (s, 1H, H_5), 10.98 (s, 1H, H_2)

^{13}C-NMR (CD_3OD, 125 MHz): δ 13.63 ($C_{4''}$), 20.36 ($C_{3''}$), 20.58 ($C_{8'}$), 24.55 ($C_{5'}$), 25.13 ($C_{4'}$), 25.38 ($C_{3'''}$), 30.44 ($C_{6'}$), 32.06 ($C_{3'}$), 32.14 ($C_{2''}$), 33.07 ($C_{2'''}$), 47.03 ($C_{4'''}$), 50.76 ($C_{1''}$), 62.70 ($C_{1'}$), 64.41 ($C_{1'''}$), 75.63 ($C_{2'}$), 122.77 (C_4), 122.98 (C_5), 123.71 (C_2), 171.26 ($C_{7'}$), 174.01 ($C_{5'''}$)

MS (ESI+, m/z): 265 [M^+, 100%], (ESI^-, m/z): 114 [$C_4H_8NCOO^-$, 35%]

Elemental analysis for $C_{20}H_{33}N_3O_4 \cdot H_2O$ calculated: C, 60.43; H, 8.87; N, 10.57. Found: C, 60.6; H, 9.0; N, 10.1

[α]$_D^{20}$ = -12.5 (c = 0.01, DMSO) for ee > 99%

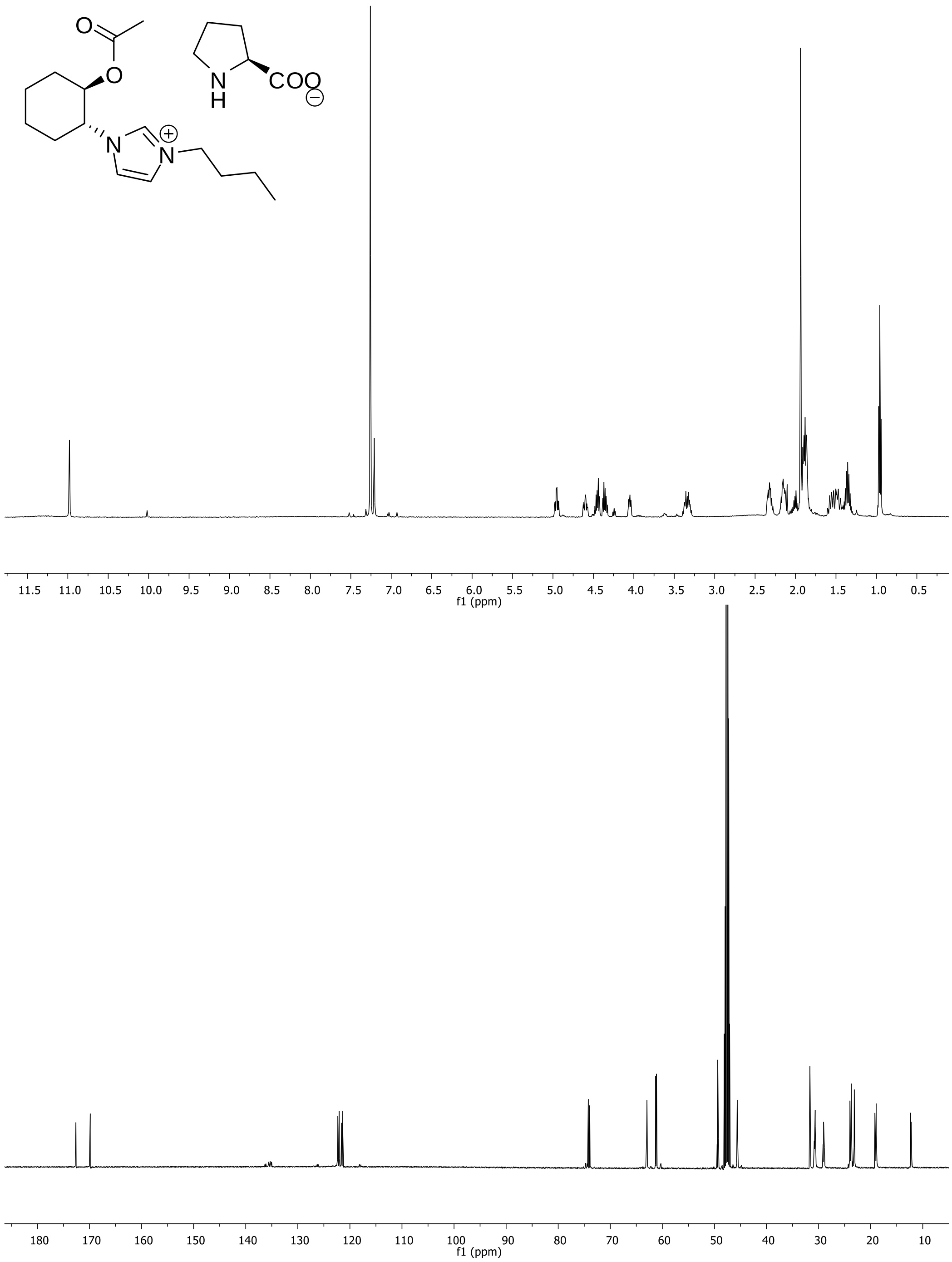

Figure S1: NMR spectra for 1-((1*R*,2*R*)-2-acetoxycyclohexyl)-3-butyl-1H-imidazol-3-ium (*S*)-pyrrolidine-2-carboxylate [(*R*,*R*)-trans-Cy6-OAc-Im-Bu-L-Pro].

3-butyl-1-((1*S*,2*S*)-2-hydroxycyclohexyl)-1H-imidazol-3-ium (*S*)-pyrrolidine-2-carboxylate [(*S*,*S*)-*trans*-Cy6-OH-Im-Bu-L-Pro]

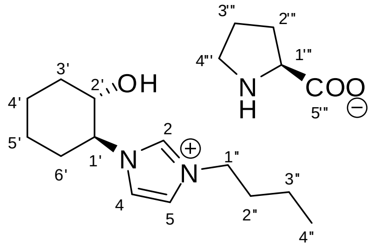

Viscous colorless liquid
Empirical formula: $C_{18}H_{31}N_3O_3$
Molecular weight: 337.46 g/mol
Melting point: 4 ºC

IR (ATR): ν 3301, 3134, 3082, 2935, 2861, 1623, 1559, 1452, 1164, 1076, 1034,955 cm^{-1}
^{1}H-NMR ($CDCl_3$, 500 MHz): δ 0.90 (t, 3H, *J* = 7.3 Hz, $H_{4''}$), 1.25-1.38 (m, 4H, $2H_{5'}$+$2H_{3''}$), 1.38-1.46 (m, 1H, $H_{3'}$), 1.52 (dc, 1H, *J* = 3.5, 13.2, 16.4 Hz, $H_{3'''}$), 1.68 (dc, 1H, *J* = 6.4, 12.6 Hz, $H_{3'''}$), 1.74-1.87 (m, 5H, $2H_{4'}$+$H_{6'}$+$2H_{2''}$), 1.89-1.99 (m, 1H, $H_{2'''}$), 2.04-2.17 (m, 2H, $H_{3'}$+$H_{6'}$), 2.24 (dt, 1H, *J* = 7.9, 15.8 Hz, $H_{2'''}$), 3.28 (t, 2H, *J* = 7.0 Hz, $H_{4'''}$), 3.57 (td, 1H, *J* = 4.4, 10.7 Hz, $H_{2'}$), 3.88-4.07 (m, 1H, $H_{1'''}$), 4.19 (t, 2H, *J* = 7.4 Hz, $H_{1''}$), 4.29-4.42 (m, 1H, $H_{1'}$), 7.17-7.23 (s, 1H, H_4), 7.25 (s, 1H, H_5), 9.96 (s, 1H, H_2)

^{13}C-NMR (DMSO, 125 MHz): δ 13.25 ($C_{4''}$), 18.77 ($C_{3''}$), 23.60 ($C_{5'}$), 23.81 ($C_{4'}$), 24.28 ($C_{3'''}$), 28.93 ($C_{6'}$), 30.81 ($C_{3'}$), 31.29 ($C_{2''}$), 34.42 ($C_{2'''}$), 45.00 ($C_{4'''}$), 48.46 ($C_{1''}$), 60.26 ($C_{1'}$), 65.18 ($C_{1'''}$), 70.91 ($C_{2'}$), 121.13 (C_4), 122.02 (C_5), 135.67 (C_2), 170.07 ($C_{5'''}$)

MS (ESI+, m/z): 223 [M^+, 100%], (ESI-, m/z): 114 [$C_4H_8NCOO^-$, 100%], 150 [$C_4H_8NCOO^-$+Cl^-, 75%]

Elemental analysis for $C_{18}H_{31}N_3O_3 \cdot 1.5H_2O$ calculated: C, 59.32; H, 9.40; N, 11.53. Found: C, 59.8; H, 7.2; N, 11.8

$[\alpha]_D^{20}$ = -21.7 (c = 0.01, DMSO) for ee > 99%

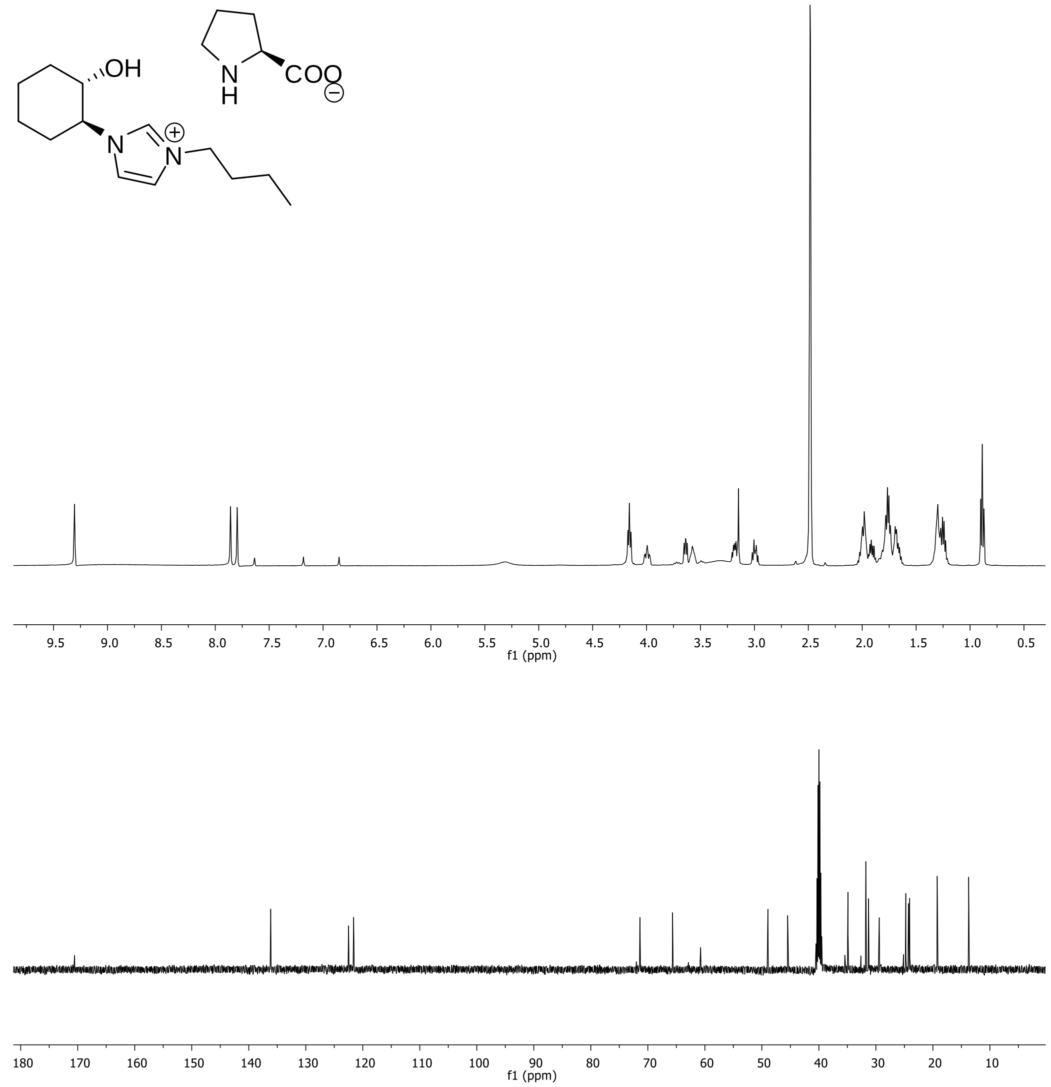

Figure S2: NMR spectra for 1-((1*S*,2*S*)-2-hydroxycyclohexyl)-3-butyl-1H-imidazol-3-ium (*S*)-pyrrolidine-2-carboxylate [(*S*,*S*)-trans-Cy6-OH-Im-Bu-L-Pro].

3-butyl-1-((1*S*,2*S*)-2-hydroxycyclohexyl)-1H-imidazol-3-ium (*R*)-pyrrolidine-2-carboxylate [(*S*,*S*)-*trans*-Cy6-OH-Im-Bu-D-Pro]

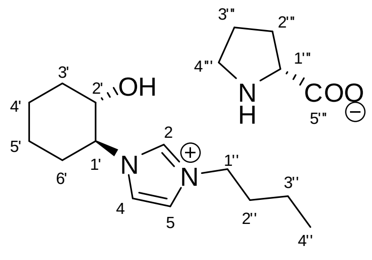

Viscous colorless solid
Empirical formula: $C_{18}H_{31}N_3O_3$
Molecular weight: 337.46 g/mol
Melting point: 2 ºC

IR (ATR): ν 3259, 3054, 2982, 2936, 2862, 1615, 1557, 1449, 1405, 1376, 1290, 1167, 1085, 1033, 952 cm^{-1}

^{1}H-NMR (DMSO, 500 MHz): δ 0.89 (3H, t, *J* = 7.4 Hz, $H_{4''}$), 1.19-1.37 (m, 5H, $2H_{5'}$+$2H_{3''}$+$H_{3'}$), 1.67 (dd, 2H, *J* = 6.3, 13.9 Hz, $H_{3'''}$), 1.78 (dt, 5H, *J* = 7.6, 22.7 Hz, $2H_{4'}$+$H_{6'}$+$2H_{2''}$), 1.91 (dd, 1H, *J* = 6.6, 12.5 Hz, $H_{2'''}$), 1.99 (dd, 3H, *J* = 7.6, 12.5 Hz, $H_{3'}$+$H_{6'}$+$H_{2'''}$), 2.93-3.06 (m, 1H, $H_{4'''}$), 3.12-3.23 (m, 1H, $H_{4'''}$), 3.57 (d, 1H, *J* = 4.3 Hz, $H_{1'''}$), 3.62 (dd, 1H, *J* = 5.7, 8.6 Hz, $H_{2'}$), 3.99 (d, 1H, *J* = 9.1 Hz, $H_{1'}$), 4.16 (t, 2H, *J* = 7.1 Hz, $H_{1''}$), 7.80 (s, 1H, H_4), 7.86 (s, 1H, H_5), 9.32 (s, 1H, H_2)

^{13}C-RMN (DMSO, 125 MHz): δ 13.25 ($C_{4''}$), 18.77 ($C_{3''}$), 23.60 ($C_{5'}$), 23.81 ($C_{4'}$), 24.28 ($C_{3'''}$), 28.93 ($C_{6'}$), 30.81 ($C_{3'}$), 31.29 ($C_{2''}$), 34.42 ($C_{2'''}$), 45.00 ($C_{4'''}$), 48.46 ($C_{1''}$), 60.26 ($C_{1'}$), 65.18 ($C_{1'''}$), 70.91 ($C_{2'}$), 121.13 (C_4), 122.02 (C_5), 135.67 (C_2), 170.07 ($C_{5'''}$)
MS (ESI+, m/z): 223 [M^+, 100%], (ESI$^-$, m/z): 114 [$C_4H_8NCOO^-$, 100%], 150 [$C_4H_8NCOO^-$+Cl^-, 75%]

Elemental analysis for $C_{18}H_{31}N_3O_3 \cdot 2H_2O$ calculated: C, 57.88; H, 9.45; N, 11.25. Found: C, 57.3; H, 9.6; N, 11.0

[α]$_D$^{20} = +20.3 (c = 0.01, DMSO) for ee > 99%

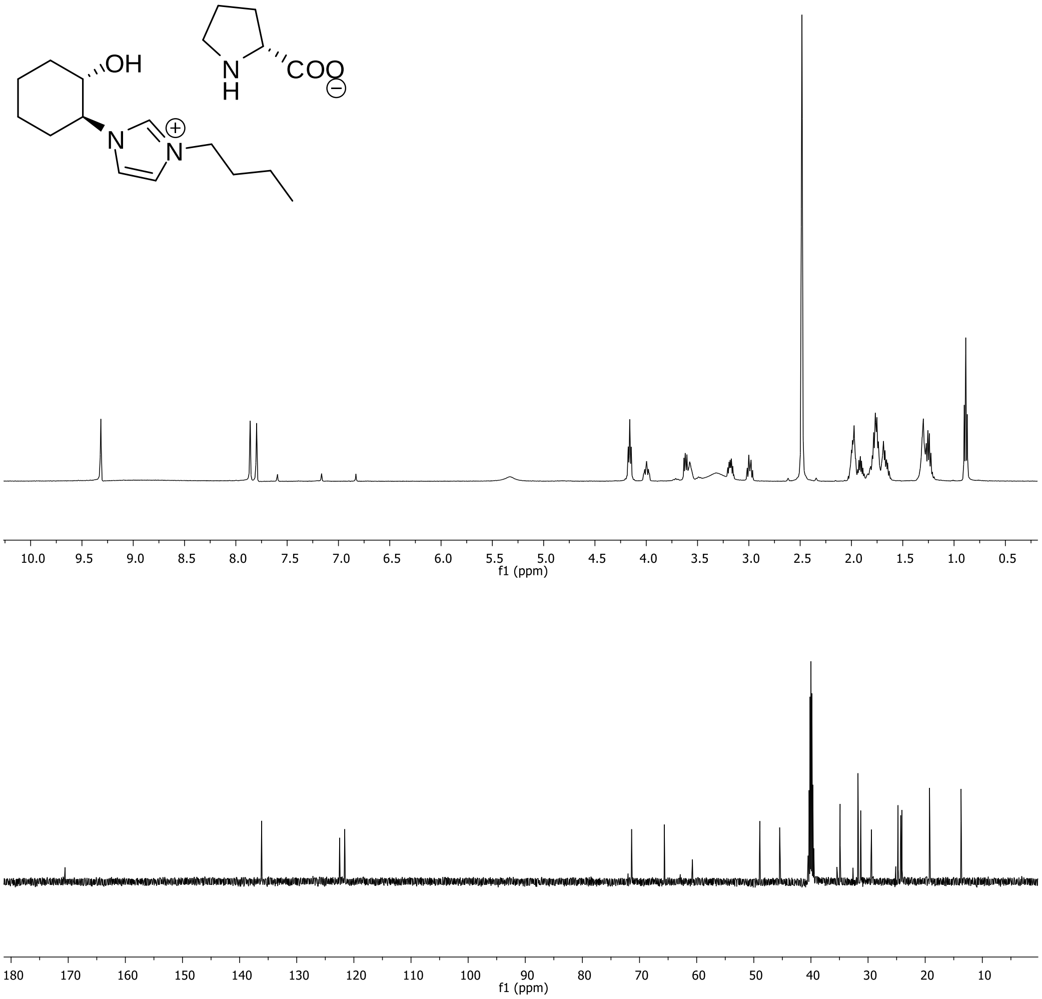

Figure S3: NMR spectra for 3-butyl-1-((1*S*,2*S*)-2-hydroxycyclohexyl)-1H-imidazol-3-ium (*R*)-pyrrolidine-2-carboxylate [(*S*,*S*)-*trans*-Cy6-OH-Im-Bu-D-Pro].

3-butyl-1-((±)-2-hydroxycyclohexyl)-1H-imidazol-3-ium (*S*)-pyrrolidine-2-carboxylate [(±)-*trans*-Cy6-OH-Im-Bu-L-Pro]

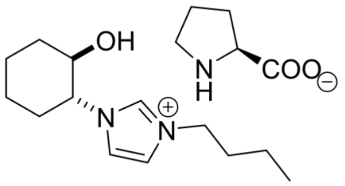

Viscous colorless liquid
Empirical formula: $C_{18}H_{31}N_3O_3$
Molecular weight: 337.46 g/mol
Melting point: 2 ºC

IR (ATR): ν 3269, 3132, 3070, 2934, 2861, 1625, 1559, 1452, 1165, 1077, 1034, 955 cm^{-1}
^{1}H-NMR ($CDCl_3$, 500 MHz): δ 0.96 (t, 3H, *J* = 7.4 Hz, $H_{4''}$), 1.31-1.50 (m, 5H, $2H_{5'}$+$2H_{3''}$+$H_{3'}$), 1.57 (dc, 1H, *J* = 3.6, 13.3, 16.4 Hz, $H_{3'''}$), 1.76 (dd, 2H, *J* = 14.1, 26.6 Hz, $H_{3'''}$), 1.82-1.94 (m, 5H, $2H_{4'}$+$H_{6'}$+$2H_{2''}$), 1.99 (dt, 1H, *J* = 6.5, 13.0 Hz, $H_{2'''}$), 2.16 (t, 2H, *J* = 14.3 Hz, $H_{3'}$+$H_{6'}$), 2.29 (dd, 1H, *J* = 7.9, 13.1 Hz, $H_{2'''}$), 3.33 (d, 2H, *J* = 3.6 Hz, $H_{4'''}$), 3.63 (td, 1H, *J* = 4.4, 10.5 Hz, $H_{2'}$), 3.99-4.10 (m, 1H, $H_{1'''}$), 4.25 (t, 2H, *J* = 7.4 Hz, $H_{1''}$), 4.35-4.49 (m, 1H, $H_{1'}$), 7.28 (d, 1H, *J* = 3.8 Hz, H_4), 7.34 (s, 1H, H_5), 10.01 (d, 1H, *J* = 4.3 Hz, H_2)

^{13}C-NMR (DMSO, 125 MHz): δ 13.25 ($C_{4''}$), 18.76 ($C_{3''}$), 23.59 ($C_{5'}$), 23.83 ($C_{4'}$), 24.26 ($C_{3'''}$), 28.92 ($C_{6'}$), 30.78 ($C_{3'}$), 31.28 ($C_{2''}$), 34.40 ($C_{2'''}$), 45.03 ($C_{4'''}$), 48.47 ($C_{1''}$), 60.36 ($C_{1'}$), 65.20 ($C_{1'''}$), 70.91 ($C_{2'}$), 121.09 (C_4), 122.04 (C_5), 135.63 (C_2), 169.90 ($C_{5'''}$)
MS (ESI+, m/z): 223 [M^+, 100%], (ESI^-, m/z): 114 [$C_4H_8NCOO^-$, 90%], 150 [$C_4H_8NCOO^-$+Cl^-, 100%]

Elemental analysis for $C_{18}H_{31}N_3O_3 \cdot H_2O$ calculated: C, 60.82; H, 9.36; N, 11.82. Found: C, 60.2; H, 8.5; N, 11.5

$[\alpha]_D^{20}$ = -17.7 (c = 0.01, DMSO) for ee > 99%

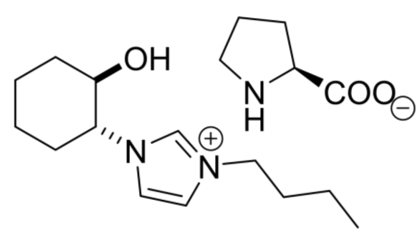

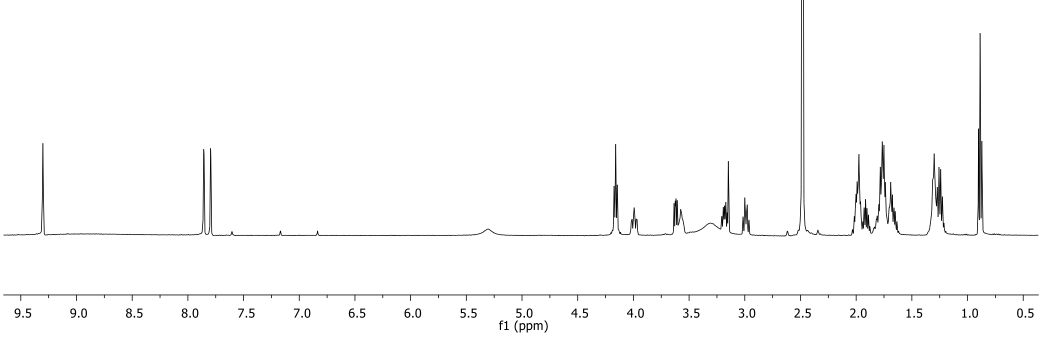

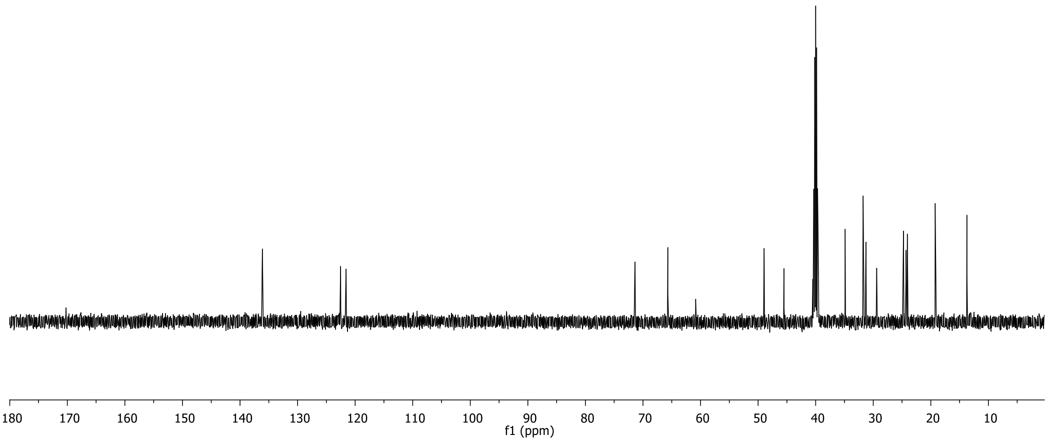

Figure S4: NMR spectra for 3-butyl-1-((±)-2-hydroxycyclohexyl)-1H-imidazol-3-ium (*S*)-pyrrolidine-2-carboxylate [(±)-*trans*-Cy6-OH-Im-Bu-L-Pro].

3-butyl-1-methyl-1H-imidazol-3-ium (*S*)-pyrrolidine-2-carboxylate [BMIM][L-Pro]

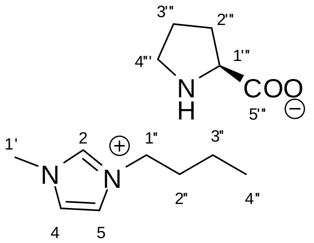

Viscous colorless solid
Empirical formula: $C_{13}H_{23}N_3O_2$
Molecular weight: 253.34 g/mol
Melting point: -33 ºC

IR (ATR): ν 3372, 3141, 3062, 2959, 2935, 2872, 1624, 1565, 1464, 1378, 1336, 1167, 1033 cm^{-1}

^{1}H-NMR (CD_3OD, 500 MHz): δ 0.99 (t, 3H, *J* = 7.4 Hz, $H_{4''}$), 1.32-1.44 (m, 2H, $H_{3''}$), 1.88 (dt, 2H, *J* = 7.5, 15.1 Hz, $H_{2''}$), 1.92-2.06 (m, 2H, $H_{3'''}$), 2.11 (td, 1H, *J* = 6.4, 13.3 Hz, $H_{2'''}$), 2.25-2.36 (m, 1H, $H_{2'''}$), 3.23-3.32 (m, 1H, $H_{4'''}$), 3.39 (dt, 1H, *J* = 6.9, 11.5 Hz, $H_{4'''}$), 3.95 (s, 3H, $H_{1'}$), 4.01 (dd, 1H, *J* = 6.1, 8.7 Hz, $H_{1'''}$), 4.24 (t, 2H, *J* = 7.3 Hz, $H_{1''}$), 7.59 (d, 1H, *J* = 1.6 Hz, H_4), 7.66 (d, 1H, *J* = 1.7 Hz, H_5), 9.01 (s, 1H, H_2)

^{13}C-NMR (CD_3OD, 125 MHz): δ 13.82 ($C_{4''}$), 20.41 ($C_{3''}$), 25.15 ($C_{3'''}$), 30.47 ($C_{2'''}$), 33.08 ($C_{2''}$), 36.60 ($C_{1'}$), 47.01 ($C_{4'''}$), 50.53 ($C_{1''}$), 62.66 ($C_{1'''}$), 123.47 (C_4), 124.76 (C_5), 138.08 (C_2), 174.12 ($C_{5'''}$)

MS (ESI+, m/z): 139 [M^+, 100%], (ESI^-, m/z): 114 [$C_4H_8NCOO^-$, 30%], 150 [$C_4H_8NCOO^-$+Cl^-, 100%]

Elemental analysis for $C_{18}H_{31}N_3O_3 \cdot H_2O$ calculated: C, 50.80; H, 9.51; N, 13.67. Found: C, 51.1; H, 8.2; N, 13.5

$[\alpha]_D^{20}$ = -31.4 (c = 0.01, DMSO) for ee > 99%

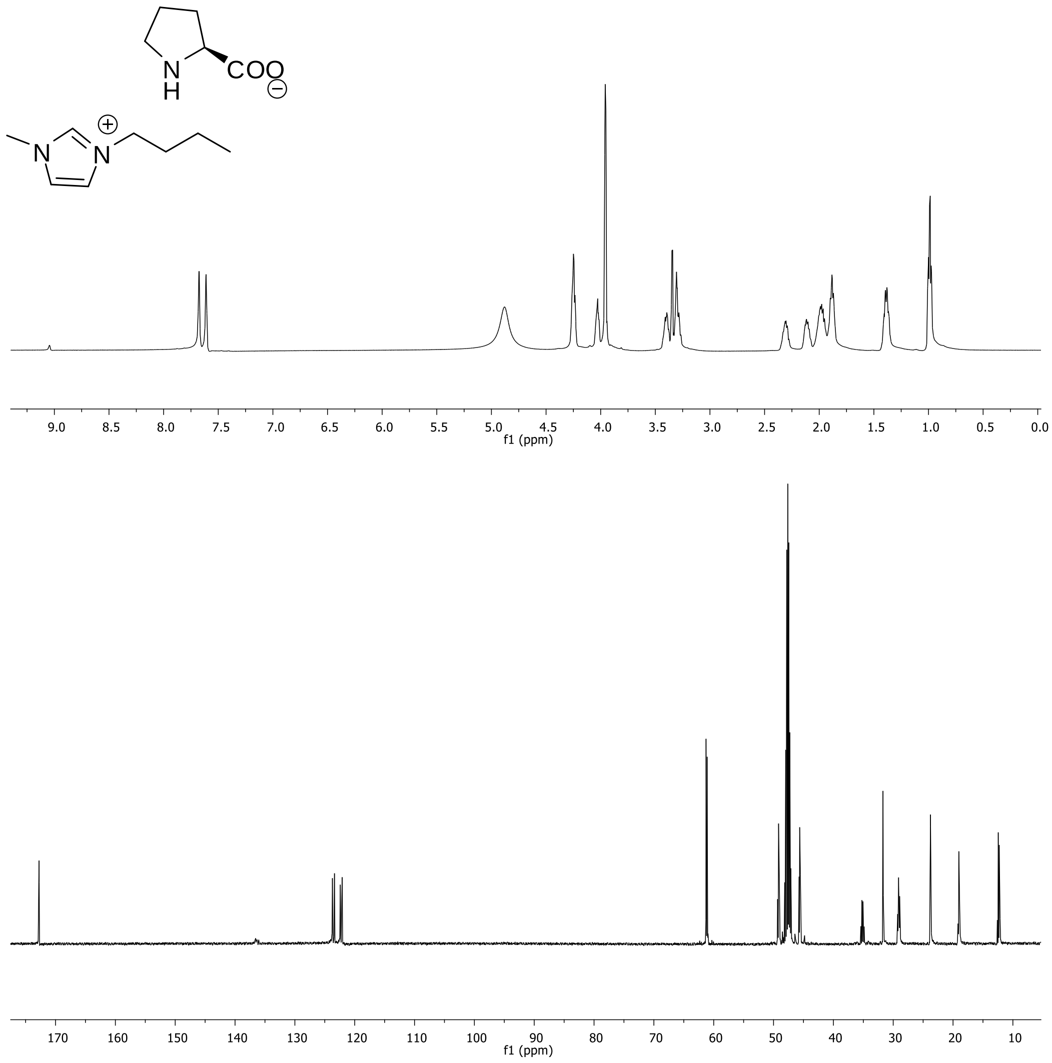

Figure S5: NMR spectra for 3-butyl-1-methyl-1H-imidazol-3-ium (*S*)-pyrrolidine-2-carboxylate [BMIM][L-Pro].

1-((1*R*,2*R*)-2-acetoxycyclopentyl)-3-butyl-1H-imidazol-3-ium (*S*)-pyrrolidine-2-carboxylate [(*R*,*R*)-*trans*-Cy5-OAc-Im-Bu-L-Pro]

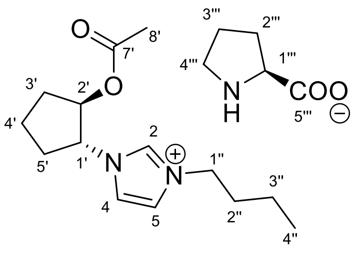

Gummy colorless solid
Empirical formula: $C_{19}H_{31}N_3O_4$
Molecular weight: 365.23 g/mol
Melting point: n.d.

IR (ATR): ν 3380, 3044, 2936, 2867, 1734, 1623, 1560, 1451, 1374, 1230, 1166, 10301, 961 cm^{-1}

^{1}H-NMR (DMSO-d$_6$, 500 MHz): δ 0.83 (t, 3H, $H_{4''}$), 1.25 (m, 2H, $H_{3''}$), 1.60-2.10 (m, 8H, $H_{3'}$+$2H_{5'}$+$2H_{4'}$+$2H_{2''}$+$H_{3'''}$), 1.96 (s, 3H, $H_{8'}$), 1.96-2.06 (m, 1H, $H_{3'''}$), 2.1-2.4 (m, 2H, $H_{3'}$+$H_{2'''}$), 2,95-3.25 (m, 3H, $H_{2'''}$, $2H_{4'''}$), 3.75 (dd, 1H, $H_{1'''}$), 4.17 (m, 2H, $H_{1''}$), 4.75 (m, 1H, $H_{2'}$), 5.18 (m, 1H, $H_{1'}$), 7.81 (s, 1H, H_4), 7.92 (s, 1H, H_5), 9.39 (s, 1H, H_2)

MS (ESI+, m/z): 251 [M$^+$], (ESI$^-$, m/z): 114 [$C_4H_8NCOO^-$]

Elemental analysis for $C_{20}H_{33}N_3O_4 \cdot H_2O$ calculated: C, 62.44; H, 8.55; N, 11.50. Found: C, 62.3; H, 8.9; N, 11.3.

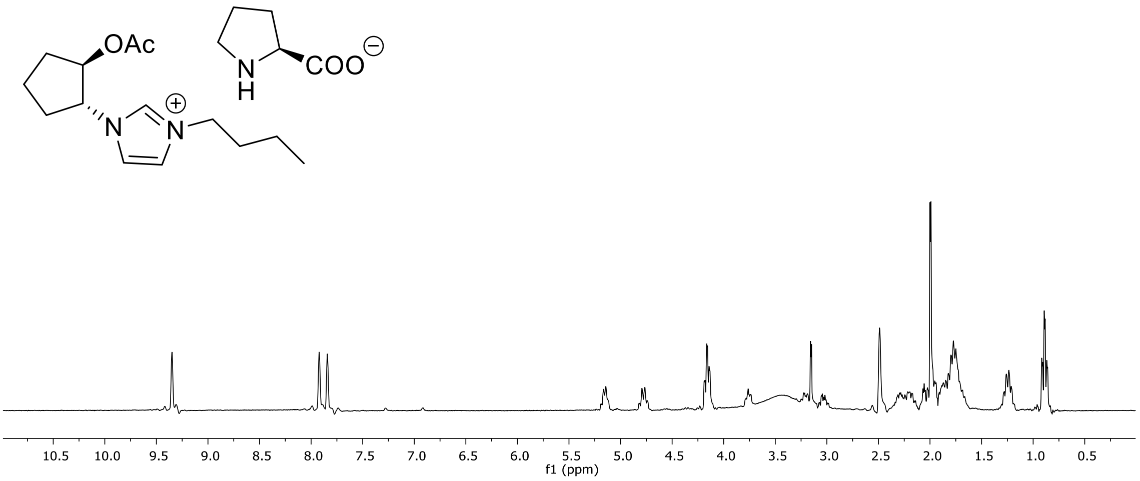

Figure S6: ^{1}H-NMR spectra NMR spectra for 1-((1*R*,2*R*)-2-acetoxycyclopentyl)-3-butyl-1H-imidazol-3-ium (*S*)-pyrrolidine-2-carboxylate [(*R*,*R*)-*trans*-Cy5-OAc-Im-Bu-L-Pro]

3-butyl-1-((1*S*,2*S*)-2-hydroxycyclopentyl)-1H-imidazol-3-ium (*S*)-pyrrolidine-2-carboxylate [(*S*,*S*)-*trans*-Cy5-OH-Im-Bu-L-Pro]

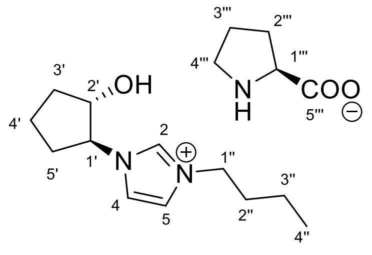

Viscous colorless liquid
Empirical formula: $C_{17}H_{29}N_3O_3$
Molecular weight: 323.22 g/mol
Melting point: n.d

IR (ATR): ν 3310, 3138, 3090, 2939, 2870, 1633, 1560, 1462, 1168, 1079, 1044,960 cm^{-1}

^{1}H-NMR (DMSO-d_6, 500 MHz): δ 0.89 (t, 3H, $H_{4''}$), 1.25 (m, 4H, $2H_{5'}$+$2H_{3''}$), 1.59 (m, 1H, $H_{3'}$), 1.61-1.83 (m, 6H, $H_{3'''}$+$H_{3'''}2H_{4'}$+$2H_{2''}$), 1.89-2.10 (m, 2H, $H_{2'''}$+$H_{3'}$), 2.24 (m, 1H, $H_{2'''}$), 3.01 (m, 2H, $H_{4'''}$), 3.25 (m, 1H, $H_{2'}$), 3.58 (m, 1H, $H_{1'''}$), 4.19 (m, 2H, $H_{1''}$), 4.42 (m, 1H, $H_{1'}$), 7.79 (s, 1H, H_4), 7.91 (s, 1H, H_5), 9.62 (s, 1H, H_2)

MS (ESI+, m/z): 209 [M$^+$], (ESI$^-$, m/z): 114 [$C_4H_8NCOO^-$].

Elemental analysis for $C_{17}H_{29}N_3O_3$, calculated: C, 60.13; H, 9.04; N, 12.99. Found: C, 59.9; H, 9.2; N, 12.9.

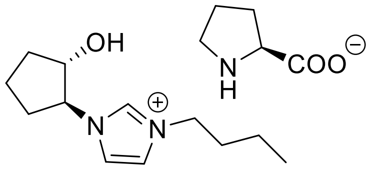

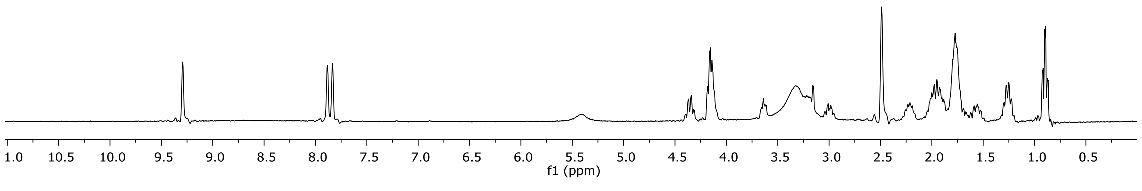

Figure S7: NMR spectra for 3-butyl-1-((1*S*,2*S*)-2-hydroxycyclopentyl)-1H-imidazol-3-ium (*S*)-pyrrolidine-2-carboxylate [(*S*,*S*)-trans-Cy5-OH-Im-Bu-L-Pro].

2. Additional catalytic experiments

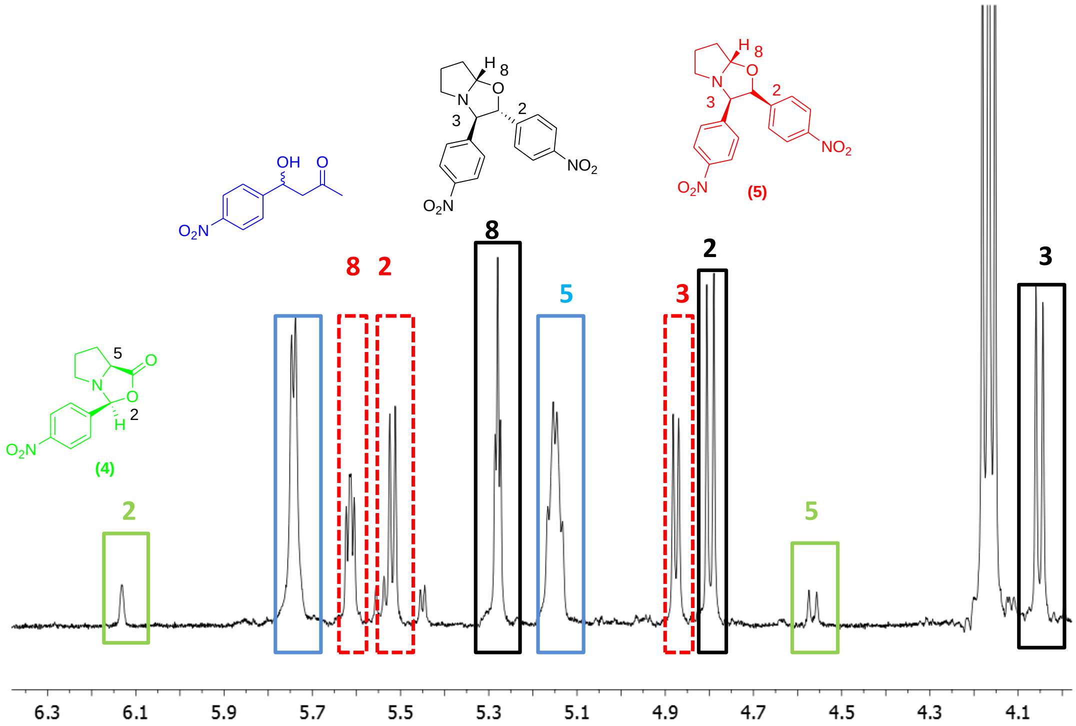

Figure S8. Representative partial ^{1}H-NMR spectrum for the crude of the reaction between acetone and *p*-nitrobenzaldehyde catalyzed by [BMIM][L-Pro] in DMSO-d_6, with the assignments for the different species formed.

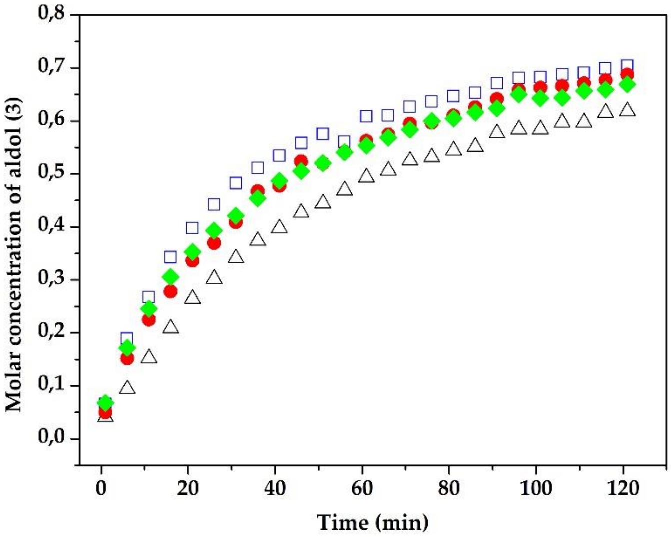

Figure S9. Evolution of the concentration of the aldol product (**3**) with time for the reaction between *p*-nitrobenzaldehyde and acetone for different catalysts using a 30:1:0.4:10 acetone:RCHO:cat:H_2O molar ratio in DMSO-d_6. Catalytic system: L-proline (□); [BMIM][L-Pro](◆); (*R*,*R*)-*trans*-Cy6-OAc-Im-Bu-L-Pro (●); L-proline:(*R*,*R*)-*trans*-Cy6-OAc-Im-Bu-Cl (1:4 molar ratio)(△).

Table S1. Results for the aldol reaction between *p*-nitrobenzaldehyde and acetone using different molar ratios of (*S*,*S*)-*trans*-Cy6-OH-Im-Bu-L-Pro:(*R*,*S*)-*cis*-Cy6-OH-Im-Bu-L-Pro (40% molar loading) at room temperature under solventless conditions. [1]

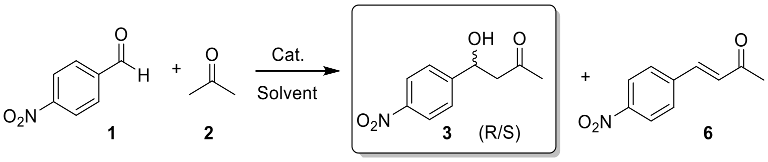

Entry	Molar ratio Cy6-OH-Im-Bu-L-Pro	Conversion (%)[2]	Selectivity (%)[2]	ee (%) [3]
1	1:0	> 99	79	43
2	0.75:0.25	> 99	96	64
3	0.5:0.5	> 99	96	62
4	0.25:0.75	> 99	95	64
5	0:1	> 99	95	64

[1] Conditions: 1:10 aldehyde(**1**):acetone(**2**) ratio, rt, 23 h, constant concentration of catalyst (0.55 M). [2] Conversion, selectivity and yield calculated by ^{1}H-NMR in the crude of the reaction. [3] Enantiomeric excess calculated by HPLC for the enantiomer *R* (major peak) [ee = (peak area (*R*) – peak area (*S*)) x 100/ total area (*R*+*S*)].

Table S2. Results for the aldol reaction between *p*-nitrobenzaldehyde and acetone using different molar ratios of (*S*,*S*)-*trans*-Cy6-OH-Im-Bu-L-Pro:(*S*,*S*)-*trans*-Cy6-OH-Im-Bu-D-Pro (40% molar loading) at room temperature under solventless conditions. [1]

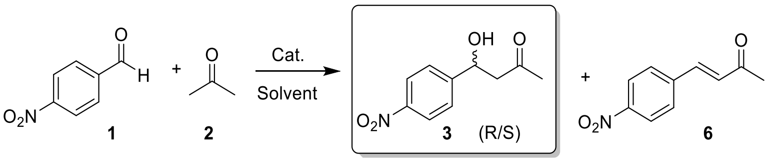

Entry	Molar ratio [(*S*,*S*)-*trans*-Cy6-OH-Im-Bu]-L-Pro:-D-Pro	Conv. (%) [2]	Selectivity (%)[2]	ee (%) [3]
1	1:0	> 99	79	40 (*R*)
2	0.875:0.125	> 99	95	21 (*R*)
3	0.75:0.25	> 99	94	18 (*R*)
4	0.625:0.375	> 99	95	20 (*R*)
5	0.5:0.5	> 99	95	10 (*S*)
6	0.375:0.625	> 99	99	20 (*S*)
7	0.25:0.75	> 99	91	28 (*S*)
8	0.125:0.875	> 99	99	37 (*S*)
9	0:1	> 99	93	50 (*S*)

[1] Conditions: 1:10 aldehyde(**1**):acetone(**2**) ratio, rt, 23 h, constant concentration of catalyst (0.55 M). [2] Conversion, selectivity and yield calculated by ^{1}H-NMR in the crude of the reaction. [3] Enantiomeric excess calculated by HPLC for the enantiomer *R* (major peak) [ee = (peak area (*R*) – peak area (*S*)) x 100/ total area (*R*+*S*)].

Table S3. Results for the aldol reaction between *p*-nitrobenzaldehyde and cyclohexanone using different prolinates (40% catalyst loading) at room temperature in DMSO. [1]

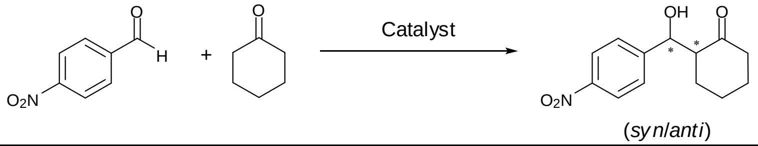

Entry	Imidazolium salts	Yield (%)[2]	Sel. *anti*:*syn*[2]	ee$_{anti}$ (%)[3]	ee$_{syn}$ (%)[3]
1	L-Pro	50	49:51	71	97
2	[BMIM][L-Pro]	53	47:53	71	97
3	(*R*,*R*)-*trans*-Cy6-OAc-Im-Bu-L-Pro	53	45:55	63	91
4	(*S*,*S*)-*trans*-Cy6-OH-Im-Bu-L-Pro	54	47:53	76	95
5	(±)-*trans*-Cy6-OH-Im-Bu-L-Pro	55	45:55	75	97

[1] Conditions: 1:10 aldehyde(**1**):cyclohexanone ratio, rt, 24 h, constant concentration of catalyst (0.05 M). [2] Selectivity and yield calculated by ^{1}H-NMR in the crude of the reaction. [3] Enantiomeric excess calculated by HPLC; the peak at as [ee = (peak area (B) – peak area (A)) x 100/ total area (A+B)] where peak (A) is the one displaying a shorter retention time in the HPLC chromatogram (for each *anti* or *syn* pair).

Table S4. Results of the aldol reaction between different aldehydes and acetone at room temperature for 24 h. [1]

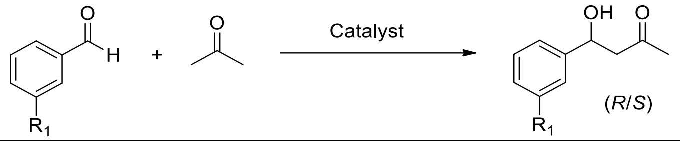

Entry	R$_1$	Catalyst	Yield (%) [2]	Selectivity (%)[2]	ee (%) [3]
1	H	L-proline	89	93	54
		(*S*,*S*)-*trans*-Cy6-OH-Im-Bu-L-Pro	51	59	81
		(*R*,*R*)-*trans*-Cy6-OAc-Im-Bu-L-Pro	31	39	79
2	Cl	L-proline	80	80	34
		(*S*,*S*)-*trans*-Cy6-OH-Im-Bu-L-Pro	71	80	59
		(*R*,*R*)-*trans*-Cy6-OAc-Im-Bu-L-Pro	66	73	62
3	OMe	L-proline	79	97	58
		(*S*,*S*)-*trans*-Cy6-OH-Im-Bu-L-Pro	58	63	66
		(*R*,*R*)-*trans*-Cy6-OAc-Im-Bu-L-Pro	50	55	71

[1] Conditions: 1:10 aldehyde:acetone(**2**) ratio, rt, 24 h, constant concentration of catalyst (0.55 M) [2] Conversion, selectivity and yield calculated by ^{1}H-NMR in the crude of the reaction. [3] Enantiomeric excess calculated by HPLC for the enantiomer *R* (major peak) [ee = (peak area (*R*) – peak area (*S*)) x 100/ total area (*R*+*S*)].

3. Characterization of aldol products [1,2,3,4]

4-hydroxy-4-(4-nitrophenyl)butan-2-one

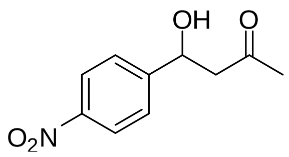

R*f*: 0.25 (hexane/ethyl acetate, 2:1)
^{1}H-RMN ($CDCl_3$, 500 MHz): δ 2.10 (s, 3H, CH_3), 2.78 (dd, 2H, *J* = 3.3, 6.6 Hz, CH_2), 3.94 (s, 1H, OH), 5.17 (dt, 1H, *J* = 3.8, 7.7 Hz, CH), 7.44 (d, 2H, Ph), 8.02 (d, 2H, *J* = 8.9 Hz, Ph)
HPLC: 36.25 min (*R*) and 41.11 min (*S*) (Chiralcel OJ, Hex/IPA (90:10), flow: 0.75 mL/min, T: 30 ºC, λ: 254 nm)

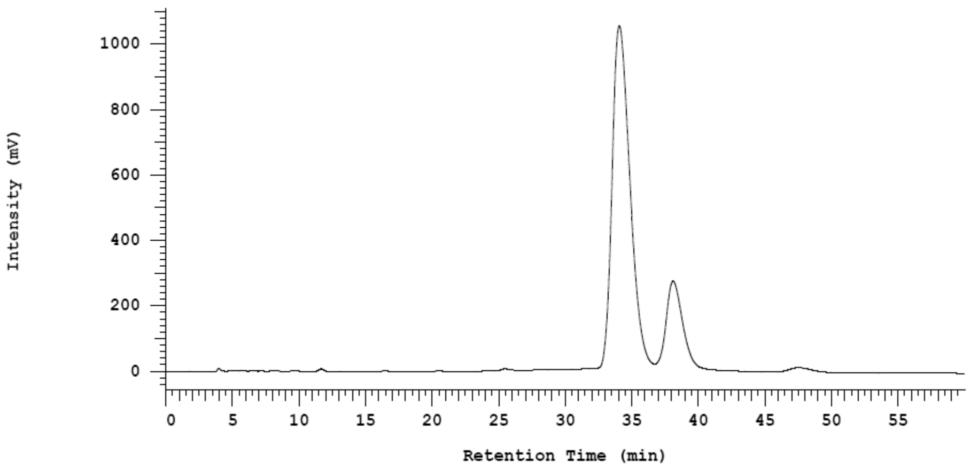

Figure S10. HPLC chromatogram for 4-hydroxy-4-(4-nitrophenyl)butan-2-one.

4-hydroxy-4-(3-methoxyphenyl)butan-2-one

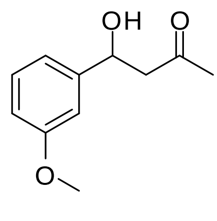

R*f*: 0.50 (hexane/ethyl acetate, 2:1)
^{1}H-RMN ($CDCl_3$, 500 MHz): δ 2.13 (s, 3H, CH_3), 2.78 (m, 2H, CH_2), 3.14 (s, 1H, OH), 3.74 (s, 3H, CH_3), 5.06 (dd, 1H, J = 3.3 Hz, J = 9.1 Hz, CH), 6.75 (m, 1H, Ph), 6.86 (m, 1H, Ph), 7.19 (m, 2H, Ph)
HPLC: 48.99 min (*R*) and 51.55 min (*S*) (Chiralcel OD-H, Hex/IPA (98:2), flow: 1 mL/min, T: 30 ºC, λ: 254 nm)

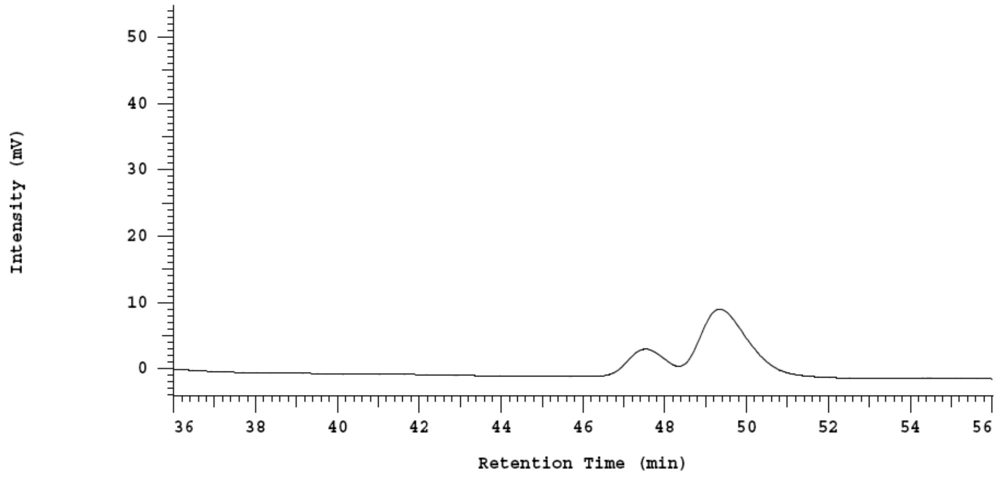

Figure S11. HPLC chromatogram for 4-hydroxy-4-(3-methoxyphenyl)butan-2-one.

4-(3-chlorophenyl)-4-hydroxybutan-2-one

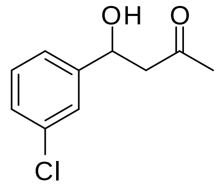

R$_f$: 0.67 (hexane/ethyl acetate, 2:1)

^{1}H-RMN ($CDCl_3$, 500 MHz): δ 2.13 (s, 3H, CH_3), 2.76 (m, 2H, CH_2), 3.31 (s, 1H, OH), 5.07 (m, 1H, CH), 7.23 (m, 4H, Ph)

HPLC: 17.02 min (*R*) and 18.95 min (*S*) (Chiralpak AD, Hex/IPA (95:5), flow: 0.750 mL/min, T: 30 ºC, *λ*: 210 nm)

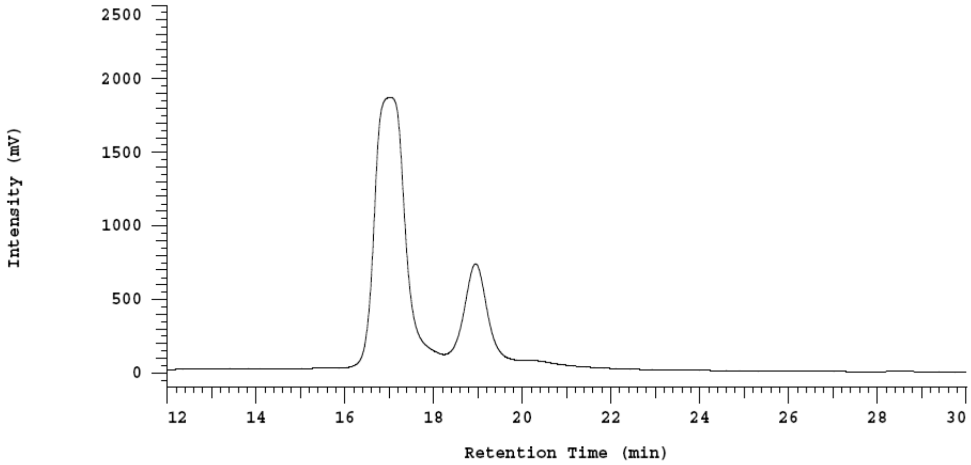

Figure S12. HPLC chromatogram for 4-(3-chlorophenyl)-4-hydroxybutan-2-one.

4-hydroxy-4-phenylbutan-2-one

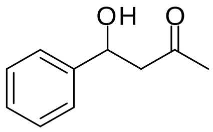

R$_f$: 0.56 (hexane/ethyl acetate, 2:1)
^{1}H-RMN ($CDCl_3$, 500 MHz): δ 2.19 (s, 3H, CH_3), 2.86 (m, 2H, CH_2), 3.25 (s, 1H, OH), 5.16 (m, 1H, CH), 7.35 (m, 5H, Ph)
HPLC: 17.23 min (*R*) and 18.57 min (*S*) (Chiralpak AD, Hex/IPA (95:5), flow: 0.750 mL/min, T: 30 ºC, *λ*: 210 nm)

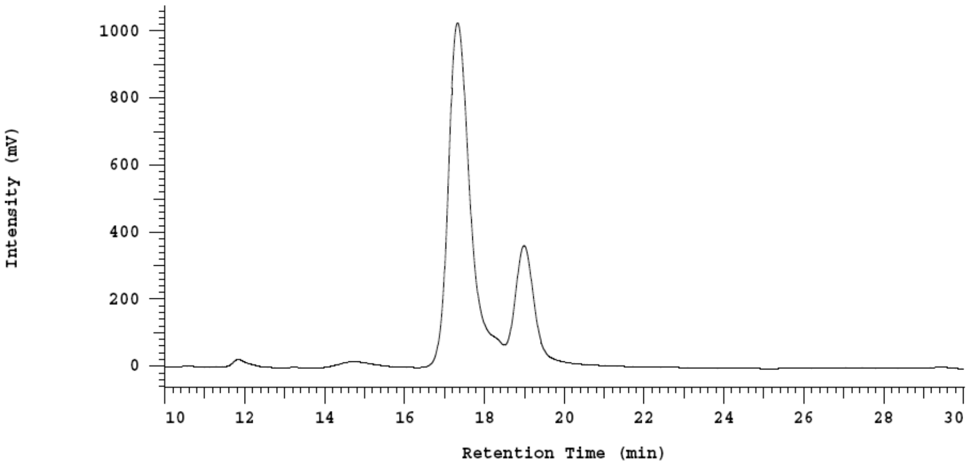

Figure S13. HPLC chromatogram for 4-hydroxy-4-phenylbutan-2-one.

2-((4-fluorophenyl)(hydroxy)methyl)cyclohexanone

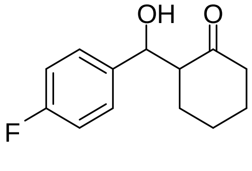

R$_f$: 0.37 (hexane/ethyl acetate, 2:1)
^{1}H-RMN ($CDCl_3$, 500 MHz): δ 1.49-1.74 (m, 6H), 2.28-2.51 (m, 3H), 3.91 (s, 1H, OH), 4.70 (d, 1H, J = 4.2 Hz, CH, *anti*), 5.22 (s, 1H, CH, *syn*), 6.94 (m, 2H, Ph), 7.20 (m, 2H, Ph)
HPLC: 17.89 min and 23.86 min (*syn*); 31.87 min and 35.76 min (*anti*) (Chiralpak AD, Hex/IPA (98:2), flow: 1 mL/min, T: 30 ºC, *λ*: 254 nm)

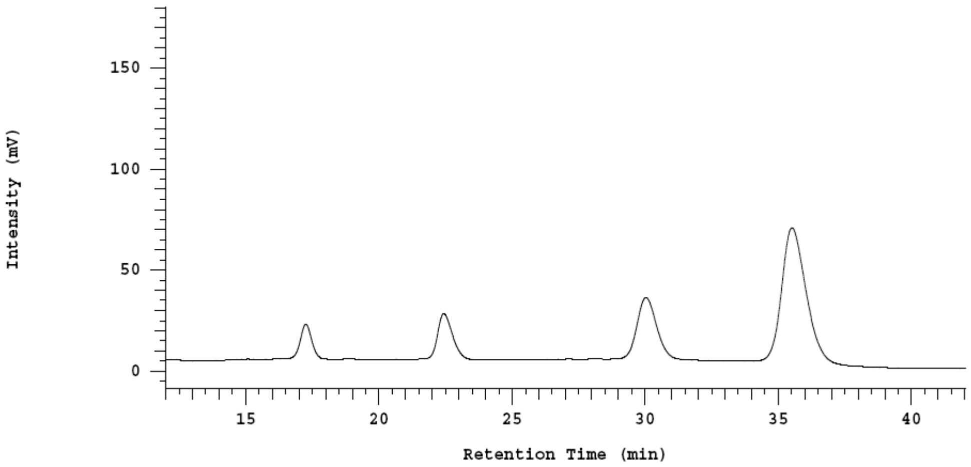

Figure S14. HPLC chromatogram for 2-((4-fluorophenyl)(hydroxy)methyl)cyclohexanone.

2-((4-chlorophenyl)(hydroxy)methyl)cyclohexanone

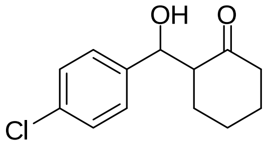

R_f: 0.41 (hexane/ethyl acetate, 2:1)
^{1}H-RMN ($CDCl_3$, 500 MHz): δ 1.53-1.67 (m, 5H), 2.34-2.56 (m, 4H), 4.03 (s, 1H, OH), 4.76 (d, 1H, J = 4.8 Hz, CH, *anti*), 5.26 (s, 1H, CH, *syn*), 7.25 (d, 2H, J = 7.3 Hz, Ph), 7.32 (d, 2H, J = 7.3 Hz, Ph)
HPLC: 21.39 min and 29.31 min (*syn*); 17.39 min and 24.67 min (*anti*) (Chiralcel OD-H, Hex/IPA (95:5), flow: 1 mL/min, T: 30 ºC, λ: 254 nm)

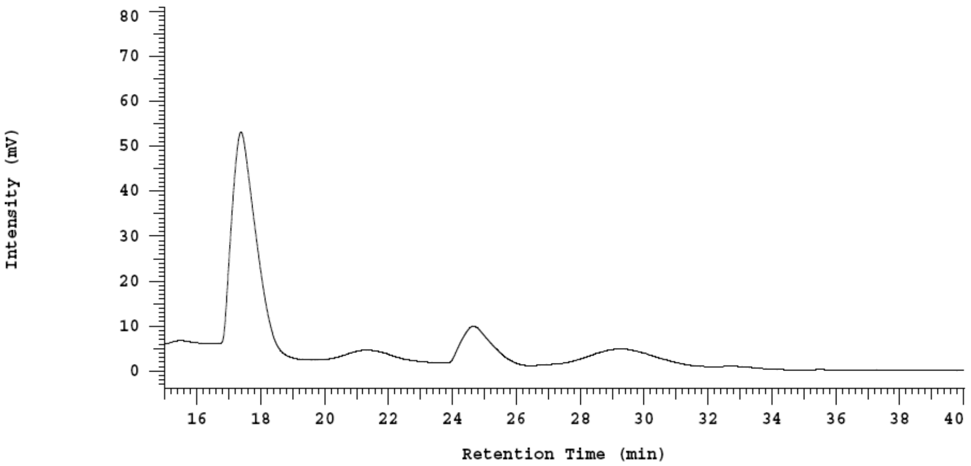

Figure S15. HPLC chromatogram for 2-((4-chlorophenyl)(hydroxy)methyl)cyclohexanone.

2-(hydroxy(4-methoxyphenyl)methyl)cyclohexanone

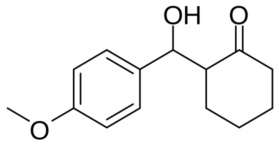

R$_f$: 0.32 (hexane/ethyl acetate, 2:1)

^{1}H-RMN ($CDCl_3$, 500 MHz): δ 1.46-1.67 (m, 5H), 2.34-2.56 (m, 4H), 3.73 (s, 3H, CH_3), 4.03 (s, 1H, OH), 4.67 (d, 1H, J = 8.9 Hz, CH, *anti*), 5.25 (s, 1H, CH, *syn*), 6.94 (m, 2H, Ph), 7.77 (m, 2H, Ph)

HPLC: 29.99 min and 37.41 min (*syn*); 56.17 min (2*S*, 1'*R*) and 59.88 min (2*R*, 1'*S*) (*anti*) (Chiralpak AD, Hex/IPA (98:2), flow: 1 mL/min, T: 30 ºC, λ: 254 nm)

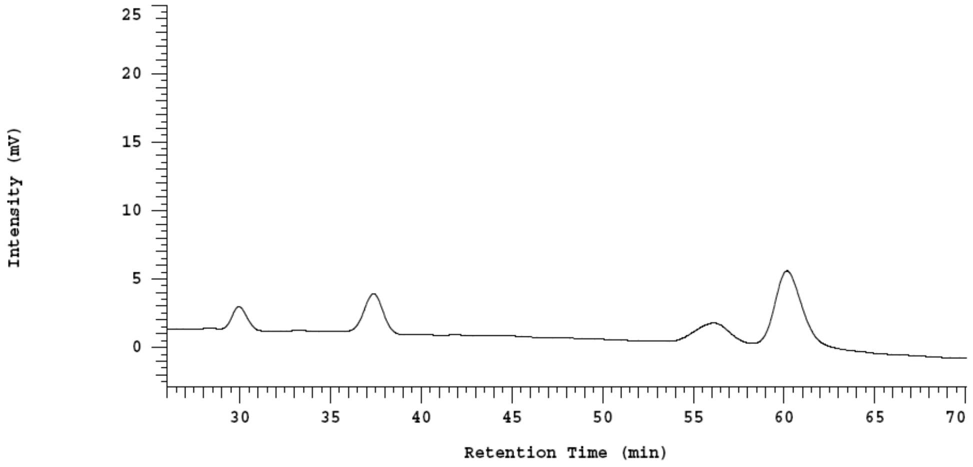

Figure S16. HPLC chromatogram for 2-(hydroxy(4-methoxyphenyl)methyl)cyclohexanone.

2-(hydroxy(phenyl)methyl)cyclohexanone

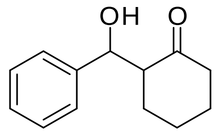

R$_f$: 0.44 (hexane/ethyl acetate, 2:1)

^{1}H-RMN ($CDCl_3$, 500 MHz): δ 1.29-1.76 (m, 6H), 2.35-2.62 (m, 3H), 3.89 (s, 1H, OH), 4.79 (d, 1H, J = 8.8 Hz, CH, *anti*), 5.32 (s, 1H, CH, *syn*), 7.32 (m, 5H, Ph)

HPLC: 12.46 min and 15.54 min (*syn*); 11.33 min and 15.13 min (*anti*) (Chiralcel OD-H, Hex/IPA (95:5), flow: 1 mL/min, T: 30 ºC, λ: 254 nm)

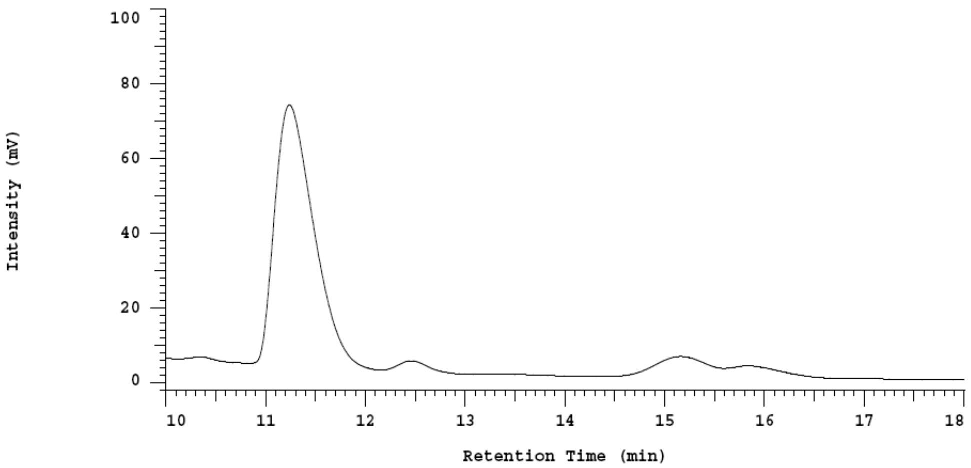

Figure S17. HPLC chromatogram for 2-(hydroxy(phenyl)methyl)cyclohexanone.

2-(hydroxy(4-nitrophenyl)methyl)cyclohexanone

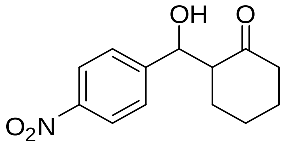

R$_f$: 0.27 (hexane/ethyl acetate, 2:1)
^{1}**H-RMN** ($CDCl_3$, 500 MHz): δ 1.32-1.78 (m, 5H), 2.26-2.53 (m, 4H), 4.02 (s, 1H, OH), 4.83 (d, 1H, J = 8.3 Hz, CH, *anti*), 5.41 (s, 1H, CH, *syn*), 7.44 (d, 2H, J = 8.5 Hz, Ph), 8.13 (d, 1H, J = 8.5 Hz, Ph)
HPLC: 16.23 min and 20.63 min (*syn*); 22.29 min (2*S*, 1'*R*) and 29.58 min (2*R*, 1'*S*) (*anti*) (Chiralpak AD, Hex/IPA (90:10), flow: 1 mL/min, T: 30 ºC, λ: 254 nm)

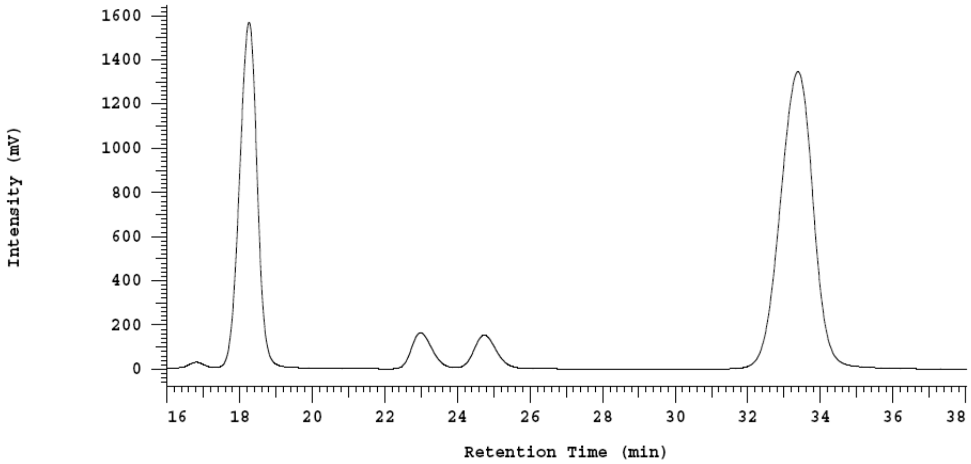

Figure S18. HPLC chromatogram for 2-(hydroxy(4-nitrophenyl)methyl)cyclohexanone.

References

1. Maya, V.; Raj, M.; Singh, V. K. Highly Enantioselective Organocatalytic Direct Aldol Reaction in an Aqueous Medium. *Org. Lett.* **2007**, *9*, 2593-2595.
2. Mase, N.; Nakai, Y.; Ohara, N.; Yoda, H.; Takabe, K.; Tanaka, F.; Barbas, C. F. Organocatalytic Direct Asymmetric Aldol Reactions in Water. *J. Am. Chem. Soc.* **2005**, *128*, 734-735.
3. Qian, Y.; Zheng, X.; Wang, Y. A Green and Efficient Asymmetric Aldol Reaction Catalyzed by a Chiral Anion Modified Ionic Liquid. *Eur. J. Org. Chem.* **2010**, 3672-3677.
4. Yang, S.-D.; Wu, L.-Y.; Yan, Z.-Y.; Pan, Z.-L.; Liang, Y.-M. A novel ionic liquid supported organocatalyst of pyrrolidine amide: Synthesis and catalyzed Claisen-Schmidt reaction. *J. Mol. Catal. A* **2007**, *268*, 107-111.
